# Evolution of community health workers: the fourth stage

**DOI:** 10.3389/fpubh.2023.1209673

**Published:** 2023-05-30

**Authors:** Nachiket Mor, Bindu Ananth, Viraj Ambalam, Aquinas Edassery, Ajay Meher, Pearl Tiwari, Vinayak Sonawane, Anagha Mahajani, Krisha Mathur, Amishi Parekh, Raghu Dharmaraju

**Affiliations:** ^1^Banyan Academy of Leadership in Mental Health, Chennai, India; ^2^Dvara Health Finance, Chennai, India; ^3^Swasthya Swaraj, Kalahandi, India; ^4^Ambuja Cement Foundation, Mumbai, India; ^5^Clinic Didi, Mumbai, India; ^6^Artificial Intelligence and Robotics Technology Park, Bengaluru, India

**Keywords:** primary care, health systems design, LMIC, cost effectiveness, access to health

## Abstract

**Introduction:**

Comprehensive primary care is a key component of any good health system. Designers need to incorporate the *Starfield* requirements of (i) a defined population, (ii) comprehensive range, (iii) continuity of services, and (iv) easy accessibility, as well as address several related issues. They also need to keep in mind that the classical British GP model, because of the severe challenges of physician availability, is all but infeasible for most developing countries. There is, therefore, an urgent need for them to find a new approach which offers comparable, possibly even superior, outcomes. The next evolutionary stage of the traditional Community health worker (CHW) model may well offer them one such approach.

**Methods:**

We suggest that there are potentially four stages in the evolution of the CHW – the health messenger, the physician extender, the focused provider, and the comprehensive provider. In the latter two stages, the physician becomes much more of an adjunct figure, unlike in the first two, where the physician is at the center. We examine the comprehensive provider stage (*stage 4*) with the help of programs that have attempted to explore this stage, using Qualitative Comparative Analysis (QCA) developed by Ragin. Starting with the 4 *Starfield* principles, we first arrive at 17 potential characteristics that could be important. Based on a careful reading of the six programs, we then attempt to determine the characteristics that apply to each program. Using this data, we look across all the programs to ascertain which of these characteristics are important to the success of these six programs. Using a *truth table*, we then compare the programs which have more than 80% of the characteristics with those that have fewer than 80%, to identify characteristics that distinguish between them. Using these methods, we analyse two global programs and four Indian ones.

**Results:**

Our analysis suggests that the global Alaskan and Iranian, and the Indian Dvara Health and Swasthya Swaraj programs incorporate more than 80% (> 14) of the 17 characteristics. Of these 17, there are 6 foundational characteristics that are present in all the six stage 4 programs discussed in this study. These include (i) *close supervision* of the CHW; (ii) *care coordination* for treatment not directly provided by the CHW; (iii) *defined referral pathways* to be used to guide referrals; (iv) *medication management* which closes the loop with patients on all the medicines that they need both immediately and on an ongoing basis (the only characteristic which needs engagement with a licensed physician); (v) *proactive care*: which ensures adherence to treatment plans; and (vi) *cost-effectiveness* in the use of scarce physician and financial resources. When comparing between programs, we find that the five essential added elements of a high-performance stage 4 program are (i) the full *empanelment* of a defined population; (ii) their *comprehensive assessment*, (iii) *risk stratification* so that the focus can be on the high-risk individuals, (iv) the use of carefully defined *care protocols*, and (v) the use of *cultural wisdom* both to learn from the community and to work with them to persuade them to adhere to treatment regimens.

## 1. Introduction

Comprehensive primary care is a key component of any good health system. There is good evidence that health systems that effectively address sources of ill health, and identify and treat diseases early in their life cycles, deliver far better health outcomes (1–3) for the same level of expenditure than do others which leave people to their own devices until they are really sick, and then use hospital-based approaches to address the problem (4). Countries such as the United Kingdom (5) and France (6) in the developed world, and Thailand (7), and Costa Rica (8) in the developing world, have built highly-effective health systems using *identify-early-address-early* approaches. In a developing country context like India, given the high untreated disease burden (9), primary care acquires particular importance. This is because any attempt to enhance hospital capacity, or the ability of Indians to pay for hospital care through subsidized private insurance or through government-financed purchasing schemes, without first building primary care, is likely to result in hospitals being overrun by people seeking care for conditions such as hypertension and diabetes that could (and should) have been dealt with far more effectively at the primary care level.

The essential principles of primary care, as articulated by Starfield (10), are well-known: (i) each primary unit must have a *defined population* that it is responsible for, (ii) it must offer a *comprehensive* range of services, (iii) it must ensure there is *continuity* of services across providers and over time, and (iv) it must be easily *accessible* to the people it is meant to serve. There are also several issues, both well-known and new, that need to be kept in mind when developing a specific design. Some of these are discussed below.

**Figure 1:**
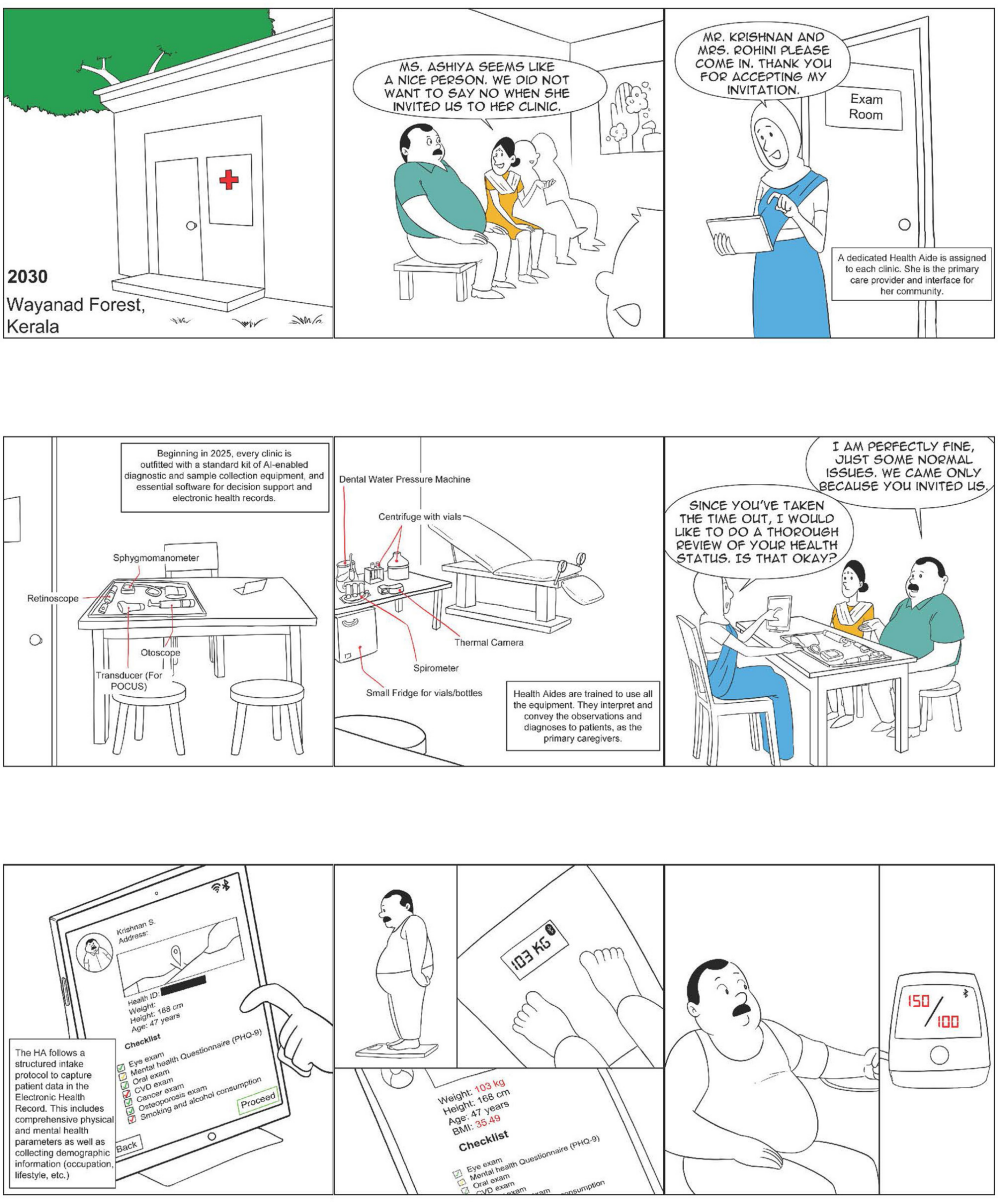
Ashiya: The CHW as a comprehensive provider (1 of 4).

**Figure 2:**
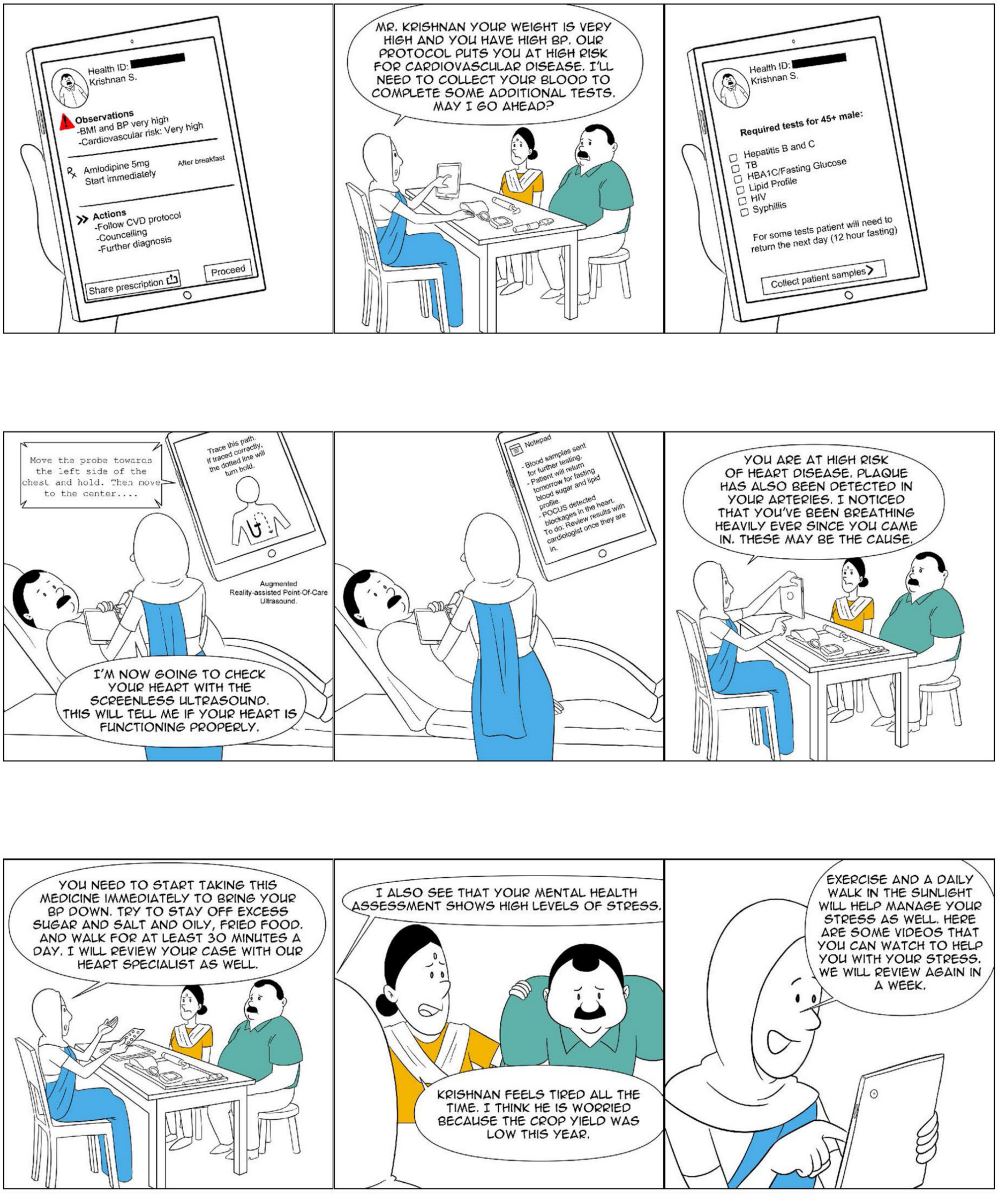
Ashiya: The CHW as a comprehensive provider (2 of 4).

**Figure 3:**
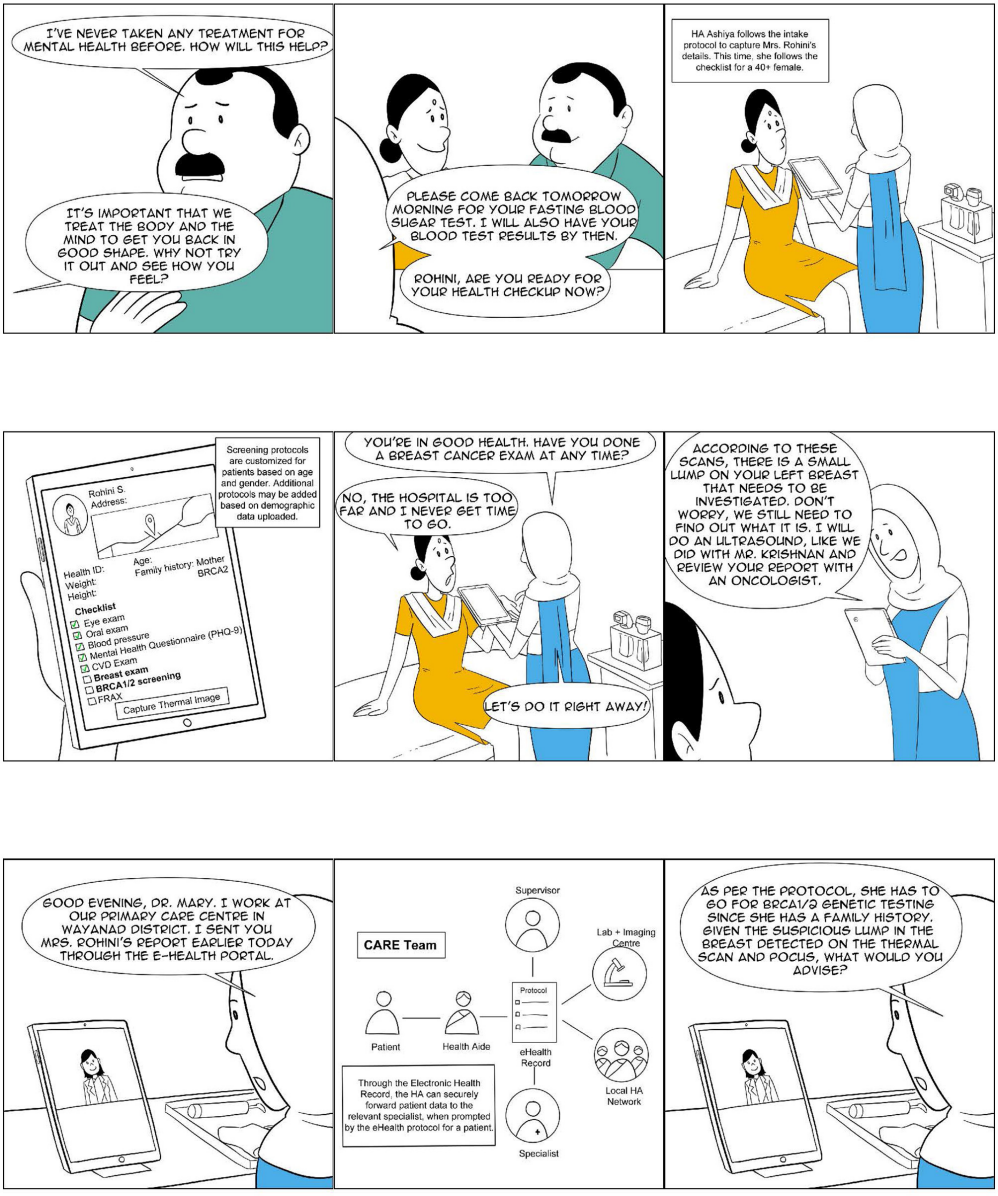
Ashiya: The CHW as a comprehensive provider (3 of 4).

**Figure 4:**
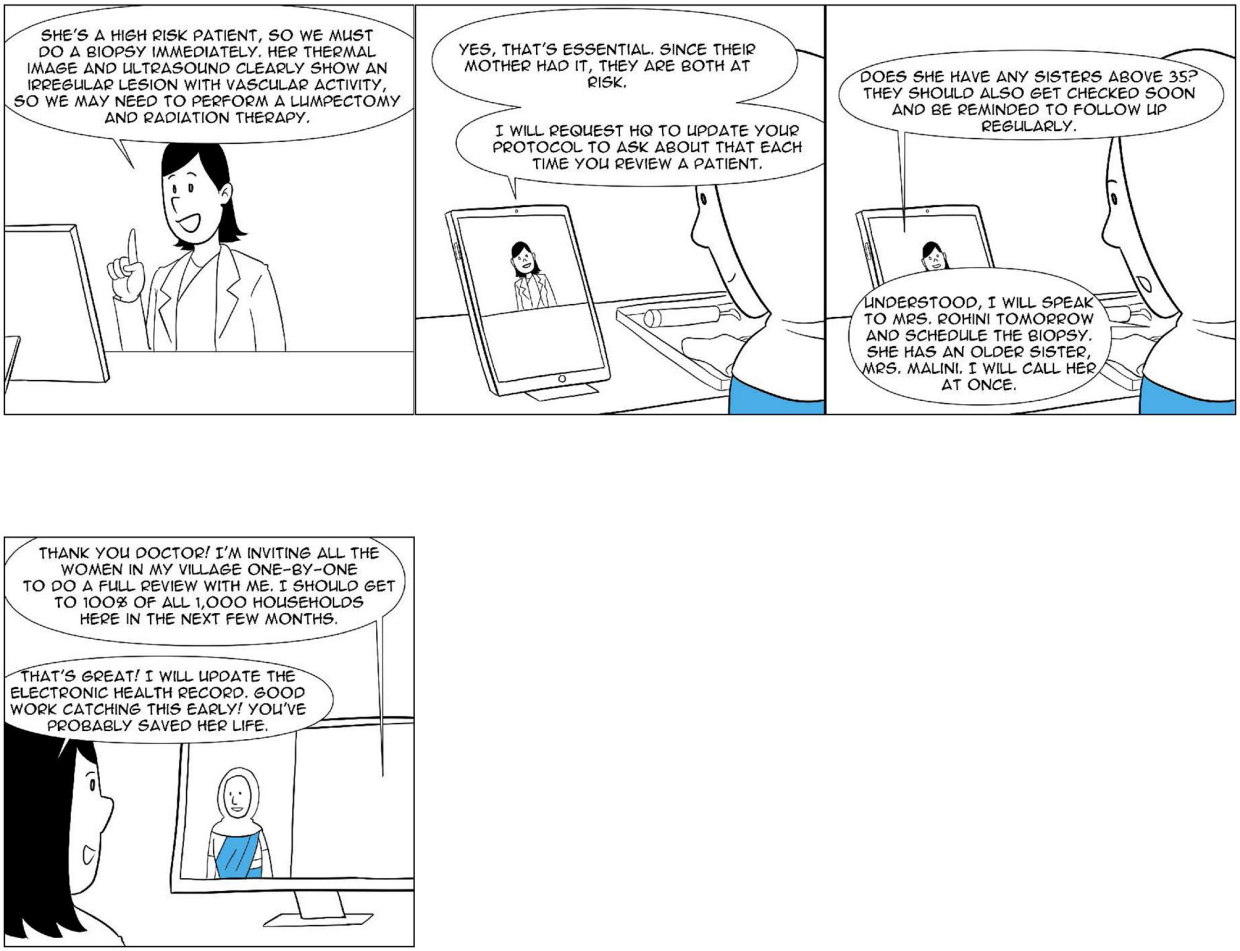
Ashiya: The CHW as a comprehensive provider (4 of 4).

1. Treatment non-adherence is a serious problem: While diagnosing the condition correctly and identifying the best way to treat it are prerequisites, a much harder problem appears to be ensuring that patients comply with the treatment recommendations. Gaudiano et al. (11) report that treatment non-adherence rates of 25% have been reported across a variety of general medical conditions, with, in the case of bipolar disorders, more than 60% of these patients [...] at least partially treatment non-adherent to medication with all the associated risks of continuing illness, relapse and exacerbation. Bhatia et al. (12) find that 20.5% of a group of children who had recovered from acute lymphoblastic leukemia (a form of blood cancer) and who needed to be on a 2-year maintenance phase that includes daily oral 6-mercaptopurine (6MP) had become non-adherent by the end of 5 months of starting this phase of their treatment, with an attendant 3.9-fold increase in the probability of relapse.
2. No health-seeking for asymptomatic conditions: As was pointed out many years ago by Hart et al. (13), patients are averse to seeking care for diseases that do not have visible symptoms. This is the case most often for chronic conditions. These need to be diagnosed and addressed well before the appearance of visible symptoms. See Figure 1 for a visualization of this in a primary care setting.
3. Social determinants of health are very important: While access to medical care is an important factor, it is now well-understood that much of the high burden of illness leading to appalling premature loss of life arises because of the immediate and structural conditions in which people are born, grow, live, work, and age (14). Primary care needs, therefore, to at least be aware of these social determinants and responsive to them, even if it is not best suited to address them in a comprehensive way.
4. The understanding of medicine is changing rapidly: Newer tools such as hand-held ultrasound machines and digital microscopy are revolutionizing what can be accomplished in primary care settings. The traditional judgement of the primary care General Physician (GP) is being supplanted by statistical algorithms such as PHQ-9 (15) for Major Depressive Disorders and B-RST (16) for genetic risks associated with breast cancer. Decision support tools and detailed protocols are making it possible to successfully screen for, diagnose, and treat a much wider range of conditions with high fidelity (17–19). See Figures 1–4 for a visualization of what a primary care clinic equipped with such tools may look like.
5. Increasing incidence of medical errors by physicians: Swann et al. find evidence which suggests that a large number of cancer patients in Britain experience avoidable delays to their diagnosis, most of which takes place in primary care settings and are attributable to misjudgement by their primary care doctors (20). In their systematic review, Stene-Larsen and Reneflot (21) find that 80% of patients who died by suicide had visited their primary care provider less than 12 months prior to their deaths, but only 31% had received any form of mental healthcare. The discrepancy between the rates of contact with primary healthcare and mental healthcare indicates that many should have received more thorough follow-up in mental healthcare (21) possibly because their primary care provider did not follow recommended screening protocols. There is evidence of overuse (in more than 50% of patient interactions) of antibiotics by licensed physicians in primary care settings in India at a rate higher than that of unlicensed medical providers (22). During COVID-19, there is similar evidence of overuse of corticosteroids by doctors leading to an outbreak of rhino-orbital-cerebral mucormycosis in India (23), and inappropriate use of Ivermectin (24) leading to toxicity and other serious side-effects. A number of these errors could be driven by an approach that privileges the certification and licensing of providers over the requirement of careful use of patient data and evidence-based protocols.
6. Physician availability is a serious challenge: There are serious shortages of doctors willing to work in primary care settings both in developing and more developed countries. These are likely to become even more severe over time (25, 26). As the proportion of older adults grows in the developed world, they are likely to attract more and more physicians from developing countries, thus exacerbating the shortages further even in developing countries like India, which have a strong domestic capacity for training physicians (27).
  There is often a view that consumers have a strong preference to be seen by physicians and that any alternate models designed to address physician shortages are likely to be rejected by them. A study of the three kinds of primary care facilities referred to as Tambon Health Promotion Hospitals (THPH), (1) main node THPHs with a doctor on rotation (THPH DR), (2) THPHs with no doctor but a nurse practitioner or registered nurse (THPH NU) and (3) THPHs staffed only by public health officers (THPH PH), was conducted in Thailand. Counter-intuitively, the results of this study showed that facilities staffed only by public health officers achieved the highest service user scores for accessibility, comprehensiveness and continuity of care in the adult patients surveyed and that the facilities staffed by doctors received the lowest scores, also falling behind nurse-led THPHs (28).
7. Cultural factors cannot be ignored: There is evidence that ignoring cultural factors can make it much harder to ensure adherence to treatment recommendations and also leads to missed opportunities to fully harness the beneficial power of many cultural practices. In tribal communities, this could mean working closely with specific rituals such as the birthing ritual *Chathi* when addressing concerns relating to maternal and child health (29) or, even more broadly, taking a holistic health systems approach in which all forms of health knowledge [are] considered diverse traditions of knowledge and not divided into binaries of modern and traditional (30). When working with young people, this could mean recognizing the power of Youth Culture and the potential of young people to become powerful agents of political and cultural change (31).
8. Rising burden of Antimicrobial Resistance: There is a rising burden of Antimicrobial Resistance (AMR) around the world (32), with developing countries like India (33) particularly adversely impacted. Any future design of primary care will need to ensure that there are careful safeguards in place against the unnecessary usage of antibiotics.

Despite all of its challenges, including those discussed above, the British GP model developed over decades by pioneers like Dr Julian Tudor Hart (34) has remained the benchmark against which all primary care systems are measured. However, in most developing countries, including India, this model, because of its reliance on trained specialist primary care providers, is all but infeasible. Recognizing this reality, many developing countries have turned to the community health worker (CHW) as offering a possible solution. Over the years, the core of the CHW, a locally recruited person who shares ethnicity, language, socioeconomic status, and life experiences with the local community (35) and is tasked with serving them, has remained unchanged. However, the breadth and complexity of tasks performed by CHWs have undergone many changes. In this paper, we refer to these changes as stages in the evolution of the CHW. We explore the question of whether the lack of availability of licensed physicians constitutes a fundamental limitation or is it, instead, a hidden opportunity to build a superior health system which is better placed to address the challenges outlined earlier, particularly at the primary care level.

## 2. Stages in the evolution of the CHW

Table 1 lists four stages in the evolution of the role of the CHW. Each of these stages is discussed in some detail below. As will become apparent from the discussion, the first two stages have, broadly, attempted to stay with the British GP model and have sought to address physician shortages using CHWs essentially as complements, with the GP performing all the functions of primary care discussed above. The next two stages have taken away from the GP all of the core primary care functions, such as managing a defined population, offering a comprehensive range of services, maintaining continuity across the referral chain, and being accessible. The role of GPs is now that of more fungible and transactional encounter-to-encounter experts, which they are called upon to play only when required. Figure 4 shows how this role could be played on an on-demand basis. It also makes the point that perhaps in such a structure, a specialist may directly need to be drawn into the discussion by the CHW. The role of the GP, as a result, may be further diminished, even if not entirely eliminated.

1. **Health Messenger**: In stage 1, the CHW, typically a part-time worker paid a small commission, is offered only very simple messaging-related tasks in a narrow field such as maternal and child health. The Indian ASHA (36) is an example of a CHW functioning at this stage. In the Indian case, even though the overall program has not been very effective (37, 38), there is evidence that whenever the ASHA has engaged with households, she has had a significant impact on their behaviors (39). Among the more successful health messengers are the Health-Development-Army volunteers in Ethiopia (40).
2. **Physician Extender**: In stage 2, the CHW, now a full-time paid worker, is assigned broader tasks which allow physicians to extend their reach beyond the clinic. The Costa Rican ATAP (41) and the Brazilian ACS (2, 42) are exemplars of such an approach.
3. **Focused Provider**: In stage 3, there is a departure from the GP model, and the CHW starts to be assigned specialized roles of high complexity, which seek to substitute for roles that the doctors are expected to play. Some examples of CHW models which work with this approach are given below.
  - In the Gadhchiroli district of Maharashtra in India, SEARCH, a local non-profit, was able to successfully demonstrate a sharp reduction in perinatal, neonatal, and infant mortality by training village health workers to diagnose and treat, among other things, neonatal sepsis with the use of injectable Gentamycin (43, 44).
  - The Ethiopians went even further and trained non-physician staff to perform Trachoma surgeries (45) and emergency obstetric surgical procedures (46) with results comparable to those of fully trained surgeons.
  - In Nepal, an intervention following such a design with a narrow focus on mental health (47, 48) showed a substantive improvement in the depression outcomes in the community, with half the patients in the intervention group experiencing more than a 50% drop in their PHQ-9 scores at endline, with about 25% of them achieving full remission (PHQ-9 < 5).
  - In Bangladesh, there are multiple categories of CHWs with distinct focused provider roles. These include Family Welfare Assistants (provision of family planning services), Health Assistants (provision of vaccinations and treatment for malaria and tuberculosis), and Community Healthcare Providers (CHCPs) who are based at Community Clinics and treat pneumonia, diarrhea, anemia, and provide booster doses of injectable contraceptives (49).

**4. Comprehensive Provider**: Despite the positive results offered by stage 3 of the evolution of the CHW, it fails to satisfy the critical primary care principle of *comprehensiveness* (10). However, the success experienced with it (and with the first two stages) opens up the possibility of a stage 4 in which CHWs are able to provide comprehensive primary care, under the remote supervision of a GP, without a GP being locally present at all times or directly interacting in-person with each patient. The two most mature expressions of this stage of the evolution of the CHW are the Alaskan *Community Health Aide* (50, 51) and the Iranian *Behvarz* (52–55).

**Table 1:** Stages in the evolution of the role of the community health worker (CHW).

**Complexity and**	**Task breadth**	
**autonomy** ↓	**Narrow**	**Broad**
Low	**Stage 1: Health Messenger**	**Stage 2: Physician Extender**
	India: ASHA	Costa Rica: ATAP
	Ethiopia: HDA	Brazil: ACS
High	**Stage 3: Focused Provider**	**Stage 4: Comprehensive Provider**
	India: SEARCH, Gentamycin injections	Iran: *Behvarz*
	Ethiopia: Trachoma surgery	Alaska: CHA
	Ethiopia: Obstetric surgery	India: Dvara *Sakhi*
	Nepal: Mental health treatment	India: Swasthya Swaraj CHP
	Bangladesh: Specialized CHWs	India: Ambuja *Sakhi*
		India: Clinic *Didi*

ASHA: Accredited Social Health Activist; HDA: Health-Development-Army; ATAP: *Asistente Tecnico en Atencion Primaria*; ACS: *Agentes Comunitarios de Saude*; SEARCH: Society for Education, Action and Research in Community Health; CHW: Community Health Worker; CHA: Community Health Aide; CHP: Community Health Practitioner.

In the following paragraphs, we review the two *comprehensive provider* programs, the Alaskan Community Health Aide Program (CHAP) and the Iranian *Behvarz* program, and a number of Indian experiments (see Table 1) at various stages of development. Through our analysis, we attempt to ascertain the key characteristics of such an approach in a developing country context such as that of India, with a particular focus on its remote rural parts. Figures 1–4 show what a stage 4 CHW as a comprehensive provider program could potentially look like in practice.

## 3. Methods

The approach we propose to use in the paper is referred to as Qualitative Comparative Analysis (QCA). It was developed by Ragin (56) and is the best method to rigorously analyse multiple programs of the type that we have here. Broadly speaking, it involves (i) developing an initial set of characteristics whose presence or absence (57) could be important in the programs being studied; (ii) identifying a set of relevant programs; (iii) developing a table of characteristics for all the programs based on a careful reading of each program to ascertain which of the initial set of characteristics are present or absent in the program; (iv) from the table of characteristics developing a truth table (58) to distinguish between these programs, and (v) determining a final set of characteristics that are the most closely associated with stage 4 CHW programs.

In this section, we discuss in some detail the initial set of characteristics that are likely to be associated with the provision of comprehensive primary care. In the Section 4, we present case studies of six programs that come closest to delivering comprehensive primary care through a community health worker. In the Section 5, we develop the *truth table* and, starting with the initial set of characteristics discussed in the Section 3, arrive at the final set of characteristics that could be most closely associated with the model of the community health worker as a comprehensive provider.

In their systematic review of the roles played by CHWs in the US, Hartzler et al. qualitatively identify 12 distinct functions (35) performed by primary care providers. They then go on to identify three overlapping clusters, which they label, Clinical Services, Community Resource Connections, and Health Education and Coaching. Using these clusters as broad categories, in Table 2 of their paper (35), they review 30 primary care interventions and indicate the different sets of functions performed by each of them. Another study of 53,000 US-based primary care practices identified 6 attributes that were significantly associated with a favorable ranking on both quality and low total annual per capita healthcare spending: (i) decision support for evidence-based medicine, (ii) risk-stratified care management, (iii) careful selection of specialists, (iv) coordination of care, (v) standing orders and protocols, and (vi) balanced physician compensation (59). Following Hartzler et al. (35) and Simon et al. (59), using Starfield's 4 principles of primary care as starting points (10), we first identify the essential components of primary care, irrespective of the specific design or method that is used to provide it.

### 3.1. Characteristics associated with *Defined Population*

1. *Empanelment*: Formal empanelment of the served population is a key critical component of primary care. As Barbara Starfield points out, it is essential that you [i.e., the primary care provider] have to know who you are responsible for, and the people have to know that they have a place to go (60). The Costa Rican model emphasizes that geographic empanelment to a specified care team encourages the formation of relationships that continue over time (41). The Joint Learning Network has recently published a report titled Empanelment: A Foundational Component of Primary Healthcare (61). A recent review of the US healthcare system by Sinsky et al. also emphasizes the importance of reorienting the system around relationships and away from an approach which sees healthcare as a series of independent encounters (i.e., transactions) that can be distributed nearly randomly among healthcare workers (62). Formal empanelment of the served population allows the practice to keep track of its patient population and to ensure their wellness over long periods of time.
2. *Comprehensive assessment*: Having empaneled a defined group carrying out a comprehensive assessment of each enrolled individual, using defined *Care protocols* would be an essential component of the onboarding process. This type of protocol-guided comprehensive assessment of each member is an effective remedy against the kinds of *errors of omission* identified by Swann et al. (20) and Stene-Larsen and Reneflot (21) which were discussed above. This could also effectively address the issue that was discussed earlier, that of patients not seeking care for diseases that do not have visible symptoms. Julian Tudor Hart, a Welsh GP, through contemporary screening for and audits of care of chronic disease and risk factors; retrospective review of computerized practice records; and comparisons of mortality and social indices with neighboring communities, was able to sharply reduce mortality in his practice relative to other neighboring practices over a period from 1964 to 1987 (13).
3. *Risk stratification*: Empanelment is merely a starting point of meeting the *Defined Population* principle. Separating these populations into different risk categories is an equally important requirement (59). Iora (63), a US-based primary care provider, every morning has a huddle, when the entire care team invests an hour discussing the health status of the clinic's population [and] prioritizes those who require the most attention and directs care around their needs guided by a *worry score* methodology, which rates each patient on a 1-to-4 scale according to their health status and needs. Patients scoring a 4 require a specific action, such as immediate outreach from a health coach. If the patient's outlook turns for the better, their *worry score* is lowered, a development celebrated by the team (64). In another example, Oak Street Health, a network of more than 80 primary care centers in medically underserved communities in the US, has successfully used machine learning-based approaches to risk stratify in its served populations (65). Risk stratification allows the practice to focus its attention sharply on those that need the most care and drive both efficiency and effectiveness.

### 3.2. Characteristics associated with *Comprehensive range*

4. *Care protocols*: *Comprehensive range* of services need to be offered in primary care settings using a set of well-defined medical protocols such as those discussed in the paper by Mor (66). In a primary care setting, individuals will often visit with multiple symptoms, some known and visible to the patient and others not. The only way to ensure that the eventual diagnosis and treatment plan is accurate and complete is to carry out a thorough examination of each patient using well-defined protocols, as well as record data for future use and analysis. This is a core function of any high-value primary care (59). The high-fidelity adherence to protocols can also help address the issues raised above relating to medical errors and the improper use of antimicrobials.
5. *Electronic instruments*: In multiple areas, there have been considerable technological advancements alongside a sharp reduction in the costs of these technologies. It is now possible to add to primary care settings mobile X-Ray machines; hand-held ultrasounds (including those without screens which make it impossible to identify the sex of an unborn child); digital microscopy, which can allow high-quality images of locally prepared slides to be analyzed by AI algorithms and remotely located pathologists; thermal imaging, which can allow the detection of abnormal cancer tissues or organisms such as worms that cause river blindness from their heat signatures; and digital photography with specialized scopes for the cervix, the mouth, the nose, and the ear. Effective primary care will need to equip staff with these technologies, which make it possible to arrive at an accurate diagnosis rapidly while avoiding making the patient travel long distances. See Figure 1 for a visualization of what a primary care clinic may look like with these instruments available for use.
6. *Computerized decision support (CDSS)*: As Simon et al. (59) find decision support for evidence-based medicine is a key attribute of high-value primary care. One of the best examples of this comes from Babylon Health (67), a UK-registered company with operations in Rwanda (68) and UK (69). In order to serve their patients more effectively, they have developed a powerful AI engine which understands and recognizes the unique way that humans express their symptoms [and] using this knowledge, combined with a patient's medical history and current symptoms [and the most updated medical protocols], it provides information [to the doctor] on possible medical conditions and common treatments [(17); square brackets contain clarificatory additions by the authors]. Some of the CDSS models already being explored in the Indian context include a locally developed tool, *Arogya Sahyog* (70), and a British CDSS widely used by GPs in New Zealand, *Arezzo* (71, 72). Figure 2 shows the CHW using such a tool to care for a patient.
7. *Comprehensive training*: Training primary care providers on critical clinical, technical, and communication skills is a key component of effective primary care. Given the availability of the protocols and tools discussed here, the training will need to shift from taking a disease-by-disease approach to one in which the primary care providers become quickly skilled at managing a wide range of conditions using protocols and the above-mentioned tools. In such an approach, while, for example, the providers will need to learn and practice the skills of phlebotomy and fine needle aspiration, the precise dosage of medicine needed to treat a condition can be looked up.
8. *Close supervision*: Given the broad breadth and complexity associated with primary care and the need for close adherence to protocols and guidelines, the strength of supervision exerts a key influence on the quality of primary care that is offered.

### 3.3. Characteristics associated with *Continuity*

9. *Electronic health records*: In order to ensure *Continuity* of care as well as to act as inputs to the provision of the other critical components of primary care that are discussed in this paper, the active use of electronic health records becomes another critical component of effective primary care.
10. *Care coordination*: While the majority of conditions faced by patients can be addressed at the primary care level, there will be a not-infrequent need for referrals, advanced care, and follow-ups (see, for example, Figure 4). Helping patients navigate this and acting as a patient advocate and guide is a key function of effective primary care.
11. *Defined referral pathways*: A key aspect of ensuring good *Care coordination*, as Simon et al. (59) also find in their study of high-value primary care, is the careful identification and development of a high-quality referral network of hospitals and specialists.

### 3.4. Characteristics associated with *Accessibility*

12. *Local clinic*: Being able to visit with the provider at a fixed location which is guaranteed to be open during certain hours and where, for example, privacy can be assured, is a key part of the *Accessibility* principle. This, where appropriate, could be just a room attached to the residence of the primary care provider and does not need to be a large hospital-like facility.
13. *Medication management*: Helping patients manage their need for medicines is a key aspect of effective primary care. Depending upon the legal requirements of the region in which the primary care facility is located, and the certification levels of the primary care providers, different approaches to ensure full legal compliance may be required. In a jurisdiction like India, a licensed physician will need to be involved to obtain a prescription for the patient. The telemedicine guidelines issued by the Indian regulator permit consultation with such a physician through electronic channels and the issuance of e-prescriptions (73). The primary care provider will need to ensure that the specified process for the issuance of these e-prescriptions is followed with high-fidelity.
14. *Proactive care*: This is another key aspect of effective primary care. Here the provider(s) do not simply wait for patients to show up at their clinics to seek treatment but go out regularly into the community to ensure the good health of the defined population that they are meant to serve. In the Cuban health system, for example, all Cubans, receive at least two home visits per year, and patients identified as high-risk can be seen as often as two times per week (74). Done well, this could prove to be the definitive remedy for the treatment non-adherence issue raised earlier.
15. *Cost-effective*: Another key aspect of *Accessibility* is the efficiency with which the primary care approach uses the two scarce resources of finance and licensed providers. Since the patient-to-licensed physician ratio has an important bearing on both critical constraints, the question of interest here is how much better than the UK average of 1,700 patients to a GP (75) each primary care approach performs. Given the severity of the constraint, the desire would be to find an approach that gets to ratios like 100,000 patients to a GP (through, for example, 20 CHWs, each managing 5,000 patients) while fulfilling the other essential functions of primary care listed here.
16. *Community recruitment*: Recruiting the staff member(s) meant to serve the population from the local community is key to ensuring *Accessibility* in part because this person is more likely to be available when needed, much less likely to be absent, much more able to communicate in and understand local languages and dialects and bring the cultural sensitivity that is so important. The locally recruited provider is also likely to be much more sensitive and responsive to the issues relating to social determinants that were discussed above.
17. *Cultural wisdom*: As mentioned above, there is evidence that ignoring cultural factors can make it much harder to ensure adherence to treatment recommendations and can also lead to missed opportunities to fully harness the beneficial power of many cultural practices (29–31). For primary care to be effective in any milieu, including tribal, urban slum, young adult, and senior citizen, it would need to bring local cultural wisdom to bear.

## 4. Data

Almost all the large community health worker programs globally are at stage 1 (health messenger) and stage 2 (physician extender). There are a few stage 3 (focused provider) programs, such as the ones mentioned earlier, but not many stage 4 programs. After a careful review of the literature and several discussions with primary care experts, we were able to find only two global programs and four in India that met the two key requirements of stage 4 that were discussed above, i.e., (i) a shift in control away from the GP to the CHW; and (ii) comprehensive care, that differentiates it from other three stages of the evolution of the CHW (also see Table 1). In this paper, we study these six programs in some depth using the methods specified earlier.

Data have been obtained from primary and secondary sources for all the six CHW programs that have been reviewed here. For the Alaskan Community Health Aide Program and the Iranian *Behvarz* program, secondary literature was used to learn about the programs. The other four programs analyzed here are all from different settings within India. Each program shared detailed descriptions of its various aspects. The detailed descriptions are all included in the Appendix (Supplementary material). The key features of the program extracted from the literature and the detailed descriptions are discussed in the following paragraphs.

This research is exempted from regulations for the protection of human subjects in research because it involves only analysis of information pre-collected for the purposes of healthcare operations of organizations from whom data have been obtained. For the purposes of this research, information is received by the investigators in such a manner that the identity of the human subjects cannot readily be ascertained directly or through identifiers linked to the subjects, the investigators have not contacted the subjects, and the investigators have not re-identified them (76).

### 4.1. Alaskan community health aide

The Alaskan Community Health Aides (CHAs) are chosen from remote rural Alaskan communities and provide a whole range of emergent, acute, and chronic care to the residents of their respective communities (77). The CHAs are required to have a minimum of high school grade mathematics and language proficiency as measured through the Test of Basic Adult Education or TABE (78). Certification as an Emergency Medical Technician (EMT) or as an Emergency Trauma Technician (ETT) has also been recently added as a requirement. Their overall training is offered in four modules of classroom training of 3–4 weeks each (79, 80). Each classroom module is followed by a supervised field training component. At the end of each such field training component, they can be certified to function as a CHA I through CHA IV and eventually as a full-fledged CHA. The formal certification using detailed standards (81) is a key part of the program.

Guided strictly by their Community Health Aide Manual or CHAM (82), among other things, CHAs provide antibiotic injections, immunization, phlebotomy, nebulizer therapy, respiratory testing, and culture collection (50). CHAs maintain their own pharmacy and provide medicines from there. CHAs use the Alaska-wide Electronic Health Record (EHR) system to input all their data. However, while the CHAM is now available as a document on the computer with hyperlinks designed to make searching easier, attempts to integrate the CHAM and the EHR into an integrated decision support system have thus far not proved successful. Additionally, while the CHA does address behavioral and dental issues when necessary, given their specialized nature, two additional cadres, the Behavioral Health Aide (BHA) and the Dental Health Aide (DHA), have been introduced, but unlike the CHA, they are still not present in every village in Alaska.

### 4.2. Iranian *Behvarz* program

Despite all the challenges that it faces, Iran has built a high-quality health system measured on all three dimensions of health systems performance, i.e., health outcomes, financial protection, and equity and responsiveness (83). With a DALY Rate [Disability Adjusted Life Year Lost per 100,000 population; (84)] of 25,164 in 2016 [*Health outcome*), an out-of-pocket expenditure proportion of 38.79%, (85)] during the same year (*financial protection*), and its extensive rural reach (*equity & responsiveness*), it is a clear outlier amongst countries with similar levels of total health expenditures (4).

A key part of Iran's success has been linked to its community health worker program, i.e., the *Behvarz* program. The program has been in operation since 1942 but started to grow significantly only after the reforms in Iran's health system in the 1980s. *Behvarz*s are typically high school graduates and undergo a 2-year training program before they are assigned to a field setting. The *Behvarz* is assigned 1,000 individuals and operates out of a rural health house (called the *Khaneh Behdasht* in Persian) in the village. She is a well-remunerated full-time employee of the government health system. Her role is spread over all the three clusters of care mentioned by Hartzler et al. (35) as being core to the provision of comprehensive primary care, i.e., Clinical Services, Community Resource Connections, and Health Education and Coaching. It includes the provision of mental health services (86), maternal and child healthcare, primary healthcare for adults, identification and follow-up for important communicable and non-communicable diseases, and symptomatic treatments along with environmental and occupational health in their area (52).

Research has shown that the role of the CHW has been central to the reduction of, among other things, cardiovascular disease and diabetes in Iran (54, 55). These are conditions in which, while diagnosis and treatment plans for most patients can easily be established using protocols, given the lifetime nature of the treatment, non-adherence is a significant challenge that needs to be overcome. Additional material on the program can be found in these papers by Doshmangir et al. (53, 87, 88).

### 4.3. Dvara health finance

Dvara Health Finance (Dvara Health) is a *Social Enterprise* promoted by the Chennai-based non-profit Dvara Holdings. Its health intervention, referred to as Neem (after the Neem tree, widely associated with good health and well-being), is a PMPM (per-member-per-month) subscription-based program in which each member is assigned to a dedicated health worker referred to as a Health Sakhi (meaning female-health-friend), who has a number of responsibilities to the program and to the members assigned to her. The *Sakhi* is a locally recruited high-school graduate or higher and is assigned to serve members who reside no more than 10 km from her home. She is trained to provide highly protocolized care and intensive follow-up to the members assigned to her. The current focus of the program is on cardiovascular diseases, but it hopes to gradually expand to cover all the disease areas.

The work of the *Sakhi* is supervised by a remotely located doctor (referred to in the program as the *Digital Doctor*) who also has the legal authority to write out a prescription for any medicines that need to be given to the member. All of the work leading up to the final handing over of the prescription is completed by the *Sakhi* using an electronic health record and guided by a computerized decision support system. The *Digital Doctor*, as legally required in India, directly engages with the patient on the *Sakhi's* mobile phone before writing out the prescription.

In its ongoing roll-out in the Satara district of Maharashtra, the Neem program has detected a high burden of cardiovascular disease in the 1,150 families, comprising about 4,000 individual members, enrolled to date. This group has been allocated among the twelve *Sakhis* who have been locally recruited and trained. The member group has been risk-stratified using detailed protocols (66), and the *Sakhis* have been engaged in intensive follow-up with the high-risk sub-groups. As a result of these efforts, while detailed results cannot yet be shared by the program, it is already known that, among others, individuals experiencing a hypertensive crisis are currently being successfully managed. See the Appendix (Supplementary material) and the Dvara Health website (89) for more details on the program.

### 4.4. Swasthya Swaraj

Swasthya Swaraj is a not-for-profit organization working toward making health a reality for the poorest and unreached. It has set up the Swasthya Swaraj Comprehensive Community Health Program in the tribal-dominated Thuamul Rampur block of the Kalahandi district of Odisha. Its program is active in 79 villages and covers a population of about 14,000 people. People from six other blocks of Kalahandi and Rayagada districts of Odisha also avail of its services. Through its health centers, it runs two programs: the *Swasthya Sathi* (health colleague) and the Community Health Practitioner (CHP) programs.

The *Swasthya Sathis* are, for the most part, tribal women chosen by the villages and receive short bursts of monthly training on responding to infectious diseases and to the needs of mothers and children. They are trained, in the case of need, to deliver low-risk babies at home and to measure temperature and blood pressure and the respiratory and fetal heart rates of infants. They are also trained to treat dehydration, offer age-adjusted doses of paracetamol and amoxicillin, and, given the high prevalence of malaria, chloroquine, and treat scabies.

The CHPs are the stage 4 CHWs trained to be comprehensive providers. unlike the *Swasthya Sathis* the CHPs are tribal women who have graduated from high school and have been trained through a 2-year full-time residential course at the Swasthya Swaraj School of Community Health Science & Practice located in Kaniguma village in the Thuamul Rampur block of the Kalahandi district of Odisha. At the end of the course, they receive a Diploma in Community Health Practice (DCHP) from Centurion University (CUTM) in Bhubaneswar, Odisha. At the end of the course and the completion of their field training, they are qualified to function autonomously as primary care providers and run a health clinic under the close supervision of a physician, who may not necessarily be on-site. See the Appendix (Supplementary material) and the Swasthya Swaraj website (90) for more details on the program.

### 4.5. Ambuja Cement Foundation

Ambuja Cement Foundation (91) is an independent development organization with a presence across twelve states in India and works toward enhancing overall prosperity in all the rural communities it serves. They have two long-standing (over 20 years of history) community health worker programs. One is referred to as the *Swasthya Sakhi* (human-health friend) and the other as the *Pashu Sakhi* (animal-health friend).

In both of these programs, health workers are hired locally, assigned to a defined population, trained broadly in medical matters, and required to offer comprehensive medical services to that defined population. Between the two *Sakhis*, the foundation has permitted and trained the *Pashu Sakhi* to offer a far deeper set of services to animals, including childbirth, vaccinations, medical treatment, and castration, than they have thus far been comfortable permitting the *Swasthya Sakhi*. Over the last several years, however, they have gradually begun that journey by opening a set of clinics for the *Swasthya Sakhi*, similar to the *Khaneh Behdasht* (Health House) used by the Iranian *Behvarz*. See the Appendix (Supplementary material) and the Ambuja Cement Foundation website (91) for more details on the program.

### 4.6. Clinic *Didi*

The Clinic *Didi* (meaning *elder-sister-at-the-clinic*) program is a systems-based intervention. It is a primary care program at the first point of contact, designed to support an Auxiliary Nurse Midwife (ANM) to deliver a range of primary care services using technology under the supervision of a remotely located doctor. The ANMs are women employed by the government, receive 18 months of training, and are placed at either a Sub Health Centre (92) or a Primary Health Centre. The Sub Health Centre (SHC) is typically a village-level clinic intended to serve a population of 5,000 individuals, while a Primary Health Centre (PHC) is a small rural government hospital serving about 30,000 people (93). The ANMs are recruited by the government through national or state-level selection processes and may not always be from the immediate areas or local communities that they are meant to serve. The Clinic *Didi* program seeks to deliver comprehensive primary care under a hub-and-spoke model with the following key components:

1. Effective technology for the SHC through a simple digital platform to record patient data and manage clinic supplies, as well as point-of-care diagnostic devices (to measure blood pressure, carry out urine analysis, and measure hemoglobin, temperature, pulse, and oxygen levels).
2. Targeted training, including protocol-based refresher training for ANMs to diagnose and treat 21 basic primary care conditions, targeted training for software and diagnostic devices, and training on clinic and patient management skills.
3. Patient and community engagement training for the ANMs, on communicating the importance of seeking timely care and wellness and healthy habits, and addressing the challenges related to faith-based and informal healers.
4. Improved operating processes for supervising the work of the ANMs, their schedules, and the clinic inventory infrastructure.

It was piloted across three SHCs and one PHC in the Jawhar block of the Palghar district of Maharashtra. The key goal of the pilot was to ascertain the ability of the Clinic *Didi* program to provide all three parts of primary care, preventive, diagnostic, and curative, under one roof and provide effective follow-up and referral services to higher levels as needed (whether the hub at PHC or larger hospitals when needed). While, over the 15 months that the pilot ran, patient-level outcomes were not measured, the program performed well in terms of customer satisfaction, efficiency, and quality parameters. See the Appendix (Supplementary material) for more details on the program.

## 5. Results

### 5.1. Across program analysis

For this analysis, we used the 17 essential characteristics described in the Section 3 and assessed which of the characteristics were met by each of the primary care programs being studied. The Alaskan CHAP and the Iranian *Behvarz* programs were scored by the first author (Mor) based on his reading of the literature on these two programs. The other programs were respectively scored by one of the authors most closely involved with the program based on their in-depth understanding of their program. Dvara *Sakhi* was scored by Ananth, Swaraj CHP by Meher, Ambuja *Sakhi* by Sonawane, and Clinic *Didi* by Mathur. In order to address the risk of bias, the first author (Mor) also scored each program independently, and the final rating used for each program was arrived at after a detailed dialogue between Mor and the person doing the rating if the ratings assigned by Mor differed from theirs.

Once the rating was completed, similar to the approach taken by Hartzler et al. (35), from the table, we then ascertained how many of the 17 essential characteristics were met by each program. This approach allowed us to simultaneously examine how close each individual program came to fully articulating all of the 17 characteristics listed above and identifying a subset of these 17 characteristics that could be considered most relevant for building the CHW as a comprehensive provider model.

Table 2 gives the results of the analysis of each program and its comparison against the 17 characteristics of primary care discussed above. Of the six programs discussed in the paper, while all the programs had a majority of the characteristics, four of them had more than 80% of them – the Alaskan CHAP, Iranian *Behvarz*, Dvara *Sakhi*, and Swaraj CHP.

**Table 2:** Characteristics of each program.

	**Programs**→	**Alaska**	**Iran**	**Dvara**	**Swaraj**	**Ambuja**	**Clinic**	**Total**	**Total**
**#**	**Characteristics** ↓	**CHAP**	***Behvarz***	***Sakhi***	**CHP**	***Sakhi***	***Didi***	**(of 6)**	**%**
	**Defined population**								
1.	Empanelment	1	1	1	1	0	1	5	83%
2.	Comprehensive assessment	1	1	1	1	1	0	5	83%
3.	Risk stratification	1	1	1	1	0	1	5	83%
	**Comprehensive range**								
4.	Care protocols	1	1	1	1	0	1	5	83%
5.	Electronic instruments	1	0	1	0	1	1	4	67%
6.	Computerized decision support (CDSS)	0	0	1	0	0	0	1	17%
7.	Comprehensive training	1	1	0	1	1	1	5	83%
8.	Close supervision	1	1	1	1	1	1	6	100%
	**Continuity**								
9.	Electronic health records	1	0	1	1	0	1	4	67%
10.	Care coordination	1	1	1	1	1	1	6	100%
11.	Defined referral pathways	1	1	1	1	1	1	6	100%
	**Accessibility**								
12.	Local clinic	1	1	0	1	1	1	5	83%
13.	Medication management	1	1	1	1	1	1	6	100%
14.	Proactive care	1	1	1	1	1	1	6	100%
15.	Cost-effective	1	1	1	1	1	1	6	100%
16.	Community recruitment	1	1	1	1	1	0	5	83%
17.	Cultural wisdom	1	1	1	1	1	0	5	83%
	Total (of 17)	16	14	15	15	12	13		
	Total (%)	94%	82%	88%	88%	71%	76%		

CHAP, Community Health Aide Program; CHP, Community Health Practitioner.

The Alaskan CHAP and the Iranian *Behvarz* program are both very mature programs with more than a 50-year history of performance and impact (50, 52). Because of these two programs, communities in remote areas have access to the best of modern medicine right where they live. The *Behvarz* have demonstrated that they have been key to controlling the NCD epidemic in rural Iran (55). Swasthya Swaraj has been present in the remote tribal district of Kalahandi in Odisha (India) for over a decade now, but the introduction of the CHP is relatively recent, as is their partnership with Centurion University in Odisha to offer a Diploma in Community Health Practice (DCHP) to the CHPs. The Dvara Health's Neem program, currently operational in the Satara district of Maharashtra (India), is also very new, and while it has shown some quick impact on conditions like hypertension, a full picture is yet to emerge.

All six programs display the following 6 characteristics suggesting that without these features, a stage 4 CHW as a comprehensive provider would not be effective:

1. *Close supervision*: This suggests that a stand-alone CHW working alone as an entrepreneur without being part of a framework is not likely to be effective.
2. *Care coordination*: This is another key primary care task which relates to helping the patient manage all of their care, including the treatment that is not provided by the CHW. Without careful coordination, patients can often get lost in the system, and all the good work of the CHW can come undone.
3. *Defined referral pathways*: indicating that no matter how skilled the CHW is, she must clearly recognize her limitations and know when and how to refer a patient to an expert.
4. *Medication management*: implying that the CHW will have to close the loop with the patient as far as pharmacotherapy is concerned and cannot simply offer advice and then leave the patient to figure things out for herself both at the time of initiating the treatment and on an ongoing basis, particularly for chronic diseases. This also underlines the concern that non-adherence is a significant issue in primary care and that the CHW is particularly well-placed to address it.
5. *Proactive care*: As discussed above, non-adherence to treatment guidelines and medication is perhaps as important an issue as arriving at a correct diagnosis. Given this reality, ensuring that patients get well and stay well will require the CHW to proactively reach out to patients and high-risk cohorts on a regular basis and not simply expect them to visit her clinic (also see Figure 1 for a visualization of this issue).
6. *Cost-effectiveness*: demonstrating that unless there is a sharp improvement in the effectiveness with which the program uses scarce financial and physician resources, there would be little point in pursuing it.

More than 80% of the programs (5 out of 6) and all the four highest-rated ones displayed the following eight characteristics, indicating that to deliver excellence, these are important characteristics to consider:

1. *Empanelment*: Given the reality that many of the diseases are often asymptomatic and it can be years before they show visible signs and symptoms, it becomes important for the CHW to ensure that every single person under her care is properly enrolled with her and is aware that she is their care provider. This is the starting point for her to proceed further.
2. *Comprehensive assessment*: Low-performing health systems, including those that deploy stage 1 to stage 3 CHWs, often take a disease-specific perspective. This is certainly useful in addressing emergencies like COVID-19 but does not provide a strong foundation on which to build a good health system. The starting point has to be a comprehensive assessment of every empaneled individual using carefully structured protocols (66).
3. *Risk stratification*: Having completed the comprehensive assessment, the next step would have to be stratifying the empaneled group into high, medium, and low-risk categories and paying particular attention to those in the high-risk cohort.
4. *Care protocols*: indicating that without a disciplined and clearly defined methodology of patient management which is adhered to strictly, it is not possible to deliver the quality of comprehensive primary care that is needed.
5. *Comprehensive training*: Even though the programs have taken very different approaches toward training, it is clear that training and certification are key to the success of any such program. The Alaskan CHAP has a much shorter and modular version of training which is very tightly focused on teaching the CHAs how to use the Community Health Aide Manual (CHAM) and training them on some key skills, such as phlebotomy and eye exams. The CHP of Swasthya Swaraj and the Iranian *Behavarz* both undergo a much longer, 2-year training program, but their training program also has a combination of classroom and field components. Dvara Health has started the training effort for its *Sakhis* with shorter focused modules and has taken an approach comparable to that of the Alaskan CHAP.
6. *Local clinic*: This does represent an added investment and expense on the part of the program. Amongst the high-performing programs, Dvara Health has not yet chosen to go in this direction. However, as discussed above, in order to improve the accessibility of the CHW and the range of services that she can offer, a local clinic which is suited to her needs, equivalent to the *Health House* of the *Behvarz*, is likely to be a key component of a full-fledged stage 4 CHW program.
7. *Community recruitment*: Recruiting CHWs from local communities is a key requirement for building a sustainable program. Otherwise, problems similar to those being encountered by other larger programs which have struggled to retain non-local recruits will emerge. Local recruitment also addresses issues such as language proficiency and building relationships with the community.
8. *Cultural wisdom*: This is an important aspect whose import and importance have not yet been fully understood. It goes well beyond simply awareness and sensitivity to local cultural issues. It involves the CHW and the overall program of which she is a part, taking a holistic health systems approach (30) which embraces local cultures and awareness and builds from them instead of working with them only in an instrumental way.

Sixty-seven percent (4 out of 6) of the programs had the following two key characteristics, with Electronic health records being used by three of the top four (75%) programs:

1. *Electronic instruments*: The is a veritable explosion of tools, including imaging solutions such as those shown in Figure 1, for the early detection of disease that can be used by the CHW. But this analysis suggests that their use is not a foundational aspect of the stage 4 CHW and can perhaps come later once the other more fundamental building blocks have been put in place.
2. *Electronic health records*: Historically, physicians like Julian Tudor Hart in the UK and the *Behvarz* in Iran have perhaps managed with manual record keeping. However, to have the full benefit of a data-rich decision-making process, there is no alternative to working with live real-time electronic health records. On top of these records modern decision support systems can be layered.

Only one program (Dvara Health) used a *Computerized decision support systems*. As tools such as Babylon have shown (17), the use of computerized systems can considerably simplify decision-making. However, as demonstrated by the Alaskan CHAs and by the South African Ideal Clinic protocols (19), it is perfectly possible to work with physical manuals even when addressing a wide range of diseases.

It is interesting to note from the Table 2 that to be successful, a program need not necessarily have both *Local clinics* and *Electronic health records*. This could potentially be because, in a local clinic, manual records could be stored. While having records is essential for all the other components, such as *Empanelment* and *Risk stratification*, these do not necessarily have to be electronic.

Similarly, while using both *Care protocols* and *Electronic instruments* is ideal (Alaska CHAP and Dvara *Sakhi*), through the careful use of well-defined protocols even without the use of electronic instruments (Iran *Behvarz*, Swaraj CHP) a successful program can be built.

### 5.2. Between program analysis

As a next step, we remove from Table 2 the characteristics that are common across all programs. In addition, we selectively eliminate a few other characteristics that are unlikely to help us explain the difference between the first four programs as a group (with > 80% of all the 17 characteristics discussed above) and the other two programs. The eliminated characteristics are discussed below:

1. Characteristics 8.*Close supervision*, 10.*Care coordination*, 11.*Defined referral pathways*, 13.*Medication management*, 14. *Proactive care*, and 15.*Cost-effective* are present in all the six programs.
2. Characteristic 6.*Computerized decision support (CDSS)* is present only in one of the first four programs and absent in all the five other programs. It will, therefore, not contribute to explaining the differences between the first four programs as a group and the other two programs.
3. Characteristics 7.*Comprehensive training* and 12.*Local clinic* are each absent only from one of the first four programs but present in all the five other programs. It will, therefore, not contribute to explaining the differences between the first four programs as a group and the other two programs.
4. Characteristics 16.*Community recruitment* and 17.*Cultural Wisdom* mirror each other. Given the fact that the only role of *Community recruitment* is facilitating *Cultural wisdom, Community recruitment* can be eliminated from the shorter list.

By doing this, from Table 2, we obtain Table 3. Table 3 now has 7 characteristics making it tractable to run a formal truth table (58) analysis using version 4.0 of the fs/QCA software program developed by Ragin and Davey (94) to build the *truth table* (Table 4). The *truth table* allows us to gain an insight into the characteristics associated with the differences between programs. From Table 4, it can be seen that all 7 characteristics are present in the Alaska CHAP and Dvara *Sakhi* programs. However, in the Iranian *Behvarz* program, characteristics 5 & 9, and in the Swasthya Swaraj CHP program, characteristic 5, are missing. The Ambuja *Sakhi* and the Clinic *Didi* programs have a larger number of characteristics from the list that are missing.

**Table 3:** Specific characteristics of each program.

	**Programs →**	**Alaska**	**Iran**	**Dvara**	**Swaraj**	**Ambuja**	**Clinic**
**#**	**Characteristics ↓**	**CHAP**	***Behvarz***	***Sakhi***	**CHP**	***Sakhi***	***Didi***
1.	Empanelment	1	1	1	1	0	1
2.	Comprehensive assessment	1	1	1	1	1	0
3.	Risk stratification	1	1	1	1	0	1
4.	Care protocols	1	1	1	1	0	1
5.	Electronic instruments	1	0	1	0	1	1
9.	Electronic health records	1	0	1	1	0	1
17.	Cultural wisdom	1	1	1	1	1	0
	Total (%)	94%	82%	88%	88%	71%	76%

CHAP, Community Health Aide Program; CHP, Community Health Practitioner.

**Table 4:** CHW truth table.

	**Programs →**	**Alaska^*a*^**				
**#**	**Characteristics ↓**	**Dvara^*b*^**	**Iran^*d*^**	**Swaraj^*d*^**	**Ambuja^*e*^**	**Clinic^*f*^**
1.	Empanelment	1	1	1	0	1
2.	Comprehensive assessment	1	1	1	1	0
3.	Risk stratification	1	1	1	0	1
4.	Care protocols	1	1	1	0	1
5.	Electronic instruments	1	0	0	1	1
9.	Electronic health records	1	0	1	0	1
17.	Cultural wisdom	1	1	1	1	0

^*a*^Alaska's Community Health Aide Program; ^*b*^Dvara *Sakhi*; ^*c*^Iranian *Behvarz*; ^*d*^Swasthya Swaraj Community Health Practitioner (CHP); ^*e*^Ambuja *Sakhi*; ^*f*^Clinic *Didi*.

From Tables 2, 4, it can be seen that there is no single characteristic that on its own is sufficient to build a high-performance program. However, if we focus only on the first four programs (Alaska, Dvara, Iran, and Swaraj), we can see that while ideally they are both useful tools, neither the use of *Electronic instruments* nor *Electronic health records* is essential to build a high-performance stage 4 CHW program. This suggests that the core elements of a high-performance program are (i) the full *Empanelment* of a defined population, (ii) their *Comprehensive assessment*, (iii) *Risk stratification* so that the focus can be on the high-risk individuals, (iv) the use of carefully defined *Care protocols*, and (v) the use of *Cultural wisdom* both to learn from the community and to work with them to persuade them to adhere to treatment regimens. If any of these five elements is missing, it will be impossible to build a high-performance program, even if they have other characteristics.

## 6. Discussion

The results presented above indicate that it is feasible to successfully develop and run a stage 4 CHW program to serve remote rural communities. The Iranians and the Alaskans have been doing so for decades, and the more recent experience of the Dvara Health, Swasthya Swaraj, Ambuja Cement Foundation, and Clinic *Didi* also provides strong support for this view. However, there are some essential elements of these programs that must be present in order for the program to succeed and others that may be introduced more gradually.

The 6 absolutely essential features are (i) close supervision of the CHW; (ii) taking responsibility for coordinating the overall healthcare of the patient; (iii) defined referral pathways for the CHW to follow so that she knows when to move away from her protocols and consult with an expert; (iv) full medication management for the patient and not simply offering advice and guidance; (v) taking a proactive approach toward managing patients and not simply waiting for them show up at her clinic; (vi) a focus on cost-effectiveness so that the program uses scarce physician and financial resources in a very parsimonious manner.

There are eight other features that are almost as important to incorporate into the program. These include (i) formal empanelment of the full cohort of patients under the CHW's care; (ii) carrying out a comprehensive assessment (protocol driven) for all the empaneled patients; (iii) stratifying them into high and low-risk cohorts so that the CHW can focus sharply on the high-risk group; (iv) the use of clearly defined care protocols; (v) comprehensive training for the CHW; (vi) establishing a locally situated clinic for the CHW from which she can operate; (vii) recruitment from the local community; (viii) fully incorporating local cultural wisdom into the program by taking a *holistic* view.

There are two features, (i) the use of modern electronic diagnostic tools; (ii) the use of electronic health records that 67% (4 out of 6) of the programs had, with Electronic health records being used by three of the top four (75%) programs. This suggests that these features would also be important to include in any high-quality program. Only one program used a Computerized decision support System, which suggests that while this could add a great deal of value, it is possible to function quite effectively even with well-structured manuals.

If we extend this analysis to examine the differences between the six programs, it becomes clear that even within stage 4 CHW programs, if there is a desire to build a truly high performing one, then without (i) carefully and fully empaneling the cohort of patients being served, (ii) assessing them comprehensively, (iii) risk stratifying them to identify those most at risk, (iv) following well-defined protocols, and (v) benefiting from local cultural wisdom, one may be able to build a good program but not a high-performance one.

It is important to note that among the Indian programs, only the Clinic *Didi* effort sought to work directly with the government. This did give the program some key advantages, such as a natural flow of patients into government clinics, and also opened up the possibility of future scale-up with the public sector. However, since the underlying design of the government health systems in India sees the CHW in more of a *Health Messenger* or a *Phyiscian Extender* role, despite the presence of an external NGO directly engaging with CHW (the ANM), the program faces a number of structural limitations such as the ratio of 5,000 individuals per ANM (all the other programs, including the large scale ones in Alaska and Iran, had fewer than 1,000 persons per CHW) and the recruitment of the ANM from outside the communities that she is assigned to serve.

## 7. Conclusions

It is well-understood that comprehensive primary care is the foundation of any good health system. However, given physician shortages worldwide, it is becoming both expensive and perhaps even infeasible to staff these efforts in a manner similar to what the British have done through their GP system. Working with locally-recruited community health workers (CHWs) offers a pathway forward that many health systems have taken. In this paper, we suggest that there have thus far possibly been four stages in the evolution of the CHW model, including (i) the health messenger; (ii) the physician extender; (iii) the focused provider; and (iv) the comprehensive provider.

We explore the fourth, the comprehensive provider, stage in some detail here through a QCA (Qualitative Comparative Analysis) of six programs which have all worked with this evolutionary stage of the CHW model. We find that there are both mature and early expressions of this model in existence and that it offers a pathway forward for building comprehensive primary care, which, while not entirely independent of the GP, uses that scarce resource in a highly parsimonious and cost-effective manner. However, there are several preconditions that such models need to satisfy if they are to perform this task in a high-quality manner We discuss these requirements in some detail in the paper.

While the focus of this paper was on the design elements of stage 4 CHW as a comprehensive provider, given the obvious attractiveness of this approach, an important question that naturally arises relates to the pathway to scaling up such a model. As the Alaskan and the Iranian programs demonstrate, there is no fundamental reason why these cannot be scaled up by an adequately resourced public sector. However, as the experience of the Clinic *Didi* program shows, for this to happen successfully, the CHW (ANM) would have to be granted a lot more autonomy, would necessarily have to be recruited only from the local community, would have to be much better equipped than she is currently, and the population ratio would have to be reduced substantially from 5,000 to 1,000 per CHW. And, for the program to be truly cost-effective at scale, the need for the entire SHC/PHC infrastructure would need to be carefully examined.

Another potential scale-up direction would be for independent primary care systems with these models to emerge, which are paid for either directly by the community using subscription-based models (as is being attempted by Dvara Health) or by larger health systems or by health insurers. The government could also decide, as is the case in the UK with the GP, to directly contract with these independent primary care systems or, alternatively, accredit and invest in them as is the case for Federally Qualified Health Centres (FQHCs) in the US (95).

## 8. Limitations

The limitations of this analysis come from its small sample size and its use of the QCA methodology to analyse them. An approach like this one does not have the rigor and validity of the gold-standard RCTs, but while the RCTs provide a good analysis of *what* the program outcomes were, it is harder to discern from them the underlying causal threads that led to those outcomes. Qualitative analysis, in general, and the QCA in particular, despite its small sample size, provides a rigorous way to delve more deeply into each program being studied and ask the *why* questions and benefit from access to the rich context associated with each program. QCA is often seen as a bridge between traditional quantitative and qualitative analysis (58). It is important to be aware, however, that there is still some debate on issues such as the reliability of causal inferences alongside more general critiques of the usefulness of the method itself for workforce policy and planning decisions.

## Data availability statement

The original contributions presented in the study are included in the article/Supplementary material, further inquiries can be directed to the corresponding author.

## Author contributions

NM conceptualized the study and wrote the first draft of the manuscript. RD and AP contributed to the design of the underlying CHW model analyzed in this study and the development of the visualization of the CHW's role, shown in Figures 1–4. BA, VA, AE, AMe, PT, VS, AMa, and KM contributed data relating to their respective CHW programs and worked closely with NM on the analysis of their programs. All authors contributed to the manuscript revision and read and approved the submitted version.
