# Supplementary material for: Evolution of community health workers: the fourth stage
Source: Front Public Health. 2023 May 30;11:1209673. doi: 10.3389/fpubh.2023.1209673 (PMC10270722; doi:10.3389/fpubh.2023.1209673)
Supplement: Supplementary file 1 [file Data_Sheet_1.PDF]

Evolution of Community Health Workers The Fourth Stage Appendix

Nachiket Mor[1], Bindu Ananth[2], Viraj Ambalam[2], Aquinas Edassery[3], Ajay Meher[3], Pearl Tiwari[4], Vinayak Sonawane[4], Anagha Mahajani[4], Krisha Mathur[5], Amishi Parekh[6], and Raghu Dharmaraju[6]

[1]Banyan Academy of Leadership in Mental Health
[2]Dvara Health Finance
[3]Swasthya Swaraj
[4]Ambuja Cement Foundation
[5]Clinic *Didi*
[6]Artificial Intelligence and Robotics Technology Park

April 29, 2023

A Dvara Health Finance

A.1 Introduction

Dvara Health Finance (DHF) is a *Social Enterprise* promoted by the Chennai-based non-profit Dvara Holdings. Its health intervention, referred to as Neem (after the Neem tree, widely associated with good health and well-being), is a PMPM (per-member-per-month) subscription-based model in which the member is assigned to a dedicated health worker, referred to as a Health Sakhi (meaning female-health-friend) who has a number of responsibilities to the program and to the members assigned to her. The *Sakhi* is a locally recruited high-school graduate or higher and is assigned to serve members living no more than 10 kilometres from her home. She is trained to provide highly protocolised care and intensive follow-up to the members assigned to her. The current focus of the program is on cardiovascular diseases.

The work of the *Sakhi* is supervised by a remotely located doctor (referred to here as the Digital Doctor) who also has the legal authority to write out a prescription for any medicines that need to be given to the member. All of the work leading up to the final handing over of the prescription is completed by the *Sakhi* using an electronic health record and guided by a computerised decision support system. The Digital Doctor, as legally required in India, directly engages with the patient on the *Sakhi's* mobile phone before writing out the prescription.

The program is in its early stages of the roll-out, has already detected a high burden of cardiovascular disease, and is now working hard to address it.

A.2 *Sakhi* roles & responsibilities

1. Attending regular training sessions for the maintenance and updating of essential health-related skills.
2. Performing baseline health assessments for Neem program members.
3. Entry of relevant data into designated data entry forms.
4. Being the first point of contact for all assigned Neem program members – each *Sakhi* is expected to manage 150-200 families assigned to her by the program.
5. Providing care to all assigned Neem program members (who, the program will ensure, will be located no more than 10 km away from the *Sakhi's* home).
6. To regularly meet with assigned Neem program members for follow-up visits and provide support and counselling proportionate to the level of attention required by each member (the greater the disease severity, the more attention needed).
7. To participate in all the relevant evaluations to maintain their Health *Sakhi* status.
8. To understand the family environments of all assigned Neem program members and be a source of support within the community.
9. Work closely with and report to the assigned Neem Digital Doctor.

A.3 Digital Doctor (DD) roles & responsibilities

The Digital Doctor referred to above is an integral part of the program and has the following roles and responsibilities.

1. *Sakhi*-related responsibilities

    (a) Oversee the training and ongoing certification of *Sakhis*. Where necessary, the DD will directly serve as the trainer.

    (b) Lead weekly meetings with the team of *Sakhis* to respond to queries, discuss and share best practices and identify members that need specific attention.

    (c) Ensure that the *Sakhis* are following the Neem protocol and code of conduct in all their interactions

    (d) Ensure that the quality of patient data entered by *Sakhi* meets expectations

    (e) Be a constant source of reference and support for program *Sakhis* and aid their overall development.

    (f) Escalate cases where patients appear to be falling off-track with respect to their mapped medical plan.

    (g) Devise care plans and manage their execution, working with *Sakhis* to achieve improved outcomes within pre-set time-frames.

    (h) Build and role-model a culture of patient-centricity, protocol adherence and continuous improvement among the *Sakhis*.

2. Patient-facing responsibilities

    (a) Provide patient-centric support and serve as a dependable source of empathy without any judgment.

    (b) Provide medical advice, prescriptions, and navigation services to patients, as envisaged by the Dvara Neem Protocol - via telemedicine platforms.

    (c) Provide ongoing periodic check-ins on patients – to ensure adherence/compliance with recommendations made.

    (d) Work with patients and their assigned *Sakhis* to identify behavioural patterns that affect behavioural change over time. Assess and address both barriers as well as drivers of change in patients.

3. Responsibilities as a member of a team of healthcare workers

    (a) Work with pilot-specific program managers; for tasks which require implementation execution; as and when required.

    (b) Interpret medical parameter data.

    (c) Facilitate referrals as required. Report cases to the Neem Program's specialist panel of doctors. Escalate cases where patients appear to be falling off-track with respect to their mapped medical plan to the attention of the physician and other clinical staff.

	Level 1	Level 2	Level 3	Level 4	Level 5
Clinical Skills	i) Screening for: HT, BMI, Vision, Oral lesions	i) First aid (non-suture/abrasion cleaning)<br>ii) Use of PoC devices<br>iii) Clinical data entry/use of NEEM 360	i) Clinical Breast Exam<br>ii) Cervical Exam	i) Taking patient histories<br>ii) Mental health screening	i) Phlebotomy<br>ii) Slide preparation
Health coaching/behavioural	i) Putting the patient at ease during screening.	i) Ability to do basic follow-up calls and initiation of treatment monitoring	Patient counselling for medication adherence	Patient counselling on diet/weight	Motivational interviewing
Leadership	Individual contributor	Trainer for new recruits: (i) Technical, and/or (ii) Soft skills/Behavioral	Supervising a team of Sakhis and coaching for performance	Can independently lead on-the-ground screening and home visit operations; including performance tracking and pay scale management.	

Figure 1: Sakhi Competency Map

A.4 Mann Deshi Bank Neem Pilot

The Neem program is being piloted with Mann Deshi Bank, a woman-owned co-operative Bank operating in the Mann taluka of the Satara district in Maharashtra. The Neem program has been live there since December 2022 and has reached the following milestones as on March 1, 2023:

- 2034 valid screenings completed
- 873 valid *Sakhi* interactions with at least one member of enrolled families (comprises80.4% of enrolled families).
- 38.2% of valid screened patients have Stage II hypertension or higher (n=778); 2.9% of valid screened patients were found to be in hypertensive crisis(n=59) (a subset of Stage II and higher).
- 6.9% of screened patients (n=141) are found to have diabetes before confirmatory testing (self-reported or Random Blood Sugar > 200 mg/dL).
- 41.6% of screened patients require confirmatory testing for diabetes (n=846).
- 34.7% of screened patients have a Body Mass Index (BMI) $\geq 25\ kg/m^2$ (n=706).
- 57.7% of screened patients have one or more of Stage II hypertension, diabetes, and BMI $\geq 25\ kg/m^2$ (n=1174).
- 387 medical prescriptions provided to these members.

NEEM
BY DVARA HEALTH FINANCE

Mann Deshi Foundation

<u>आरोग्य सखी फील्ड किट</u>

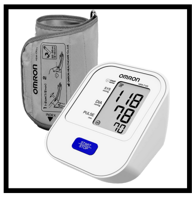

बी पी मशीन: रक्तदाब तपासण्या साठी वापरले जाते

पल्स ऑक्सिमीटर: पल्स रेट आणि रक्ता तील ऑक्सिजनचे प्रमाण मोजण्या साठी वापरले जाते

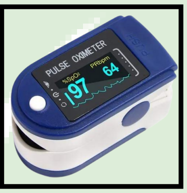

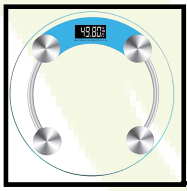

वजनाचा स्केल: जेव्हा रुग्ण त्यावर उभा असतो तेव्हा शरीराचे वजन मोजण्यासाठी वापरले जाते

थर्मामीटर: शरीरा चे तापमान मोजण्या साठी वापरले जाते

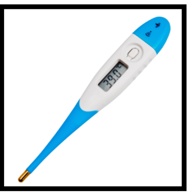

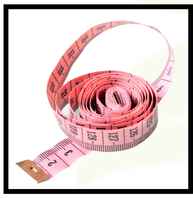

मापन टेप: उंची (सेंटीमीटरमध्ये) आणि कंबरेचा घेर (इंचांमध्ये) लक्षात ठेवण्यासाठी वापरला जातो.

ग्लुकोमीटर : हे यंत्र रक्तातील साखर मोजते

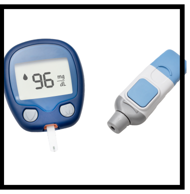

Figure 2: Sakhi Field Kit (Marathi) (Page 1/3)

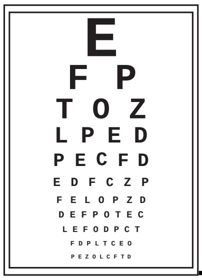

स्नेलन चार्ट: रुग्णाची दृष्टी किती चांगली आहे हे तपासण्यासाठी (जवळ किंवा दूर; अंतर))

हृदयरोगासाठी WHO जोखीम तक्ता

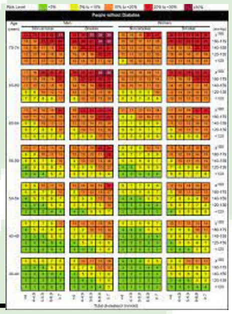

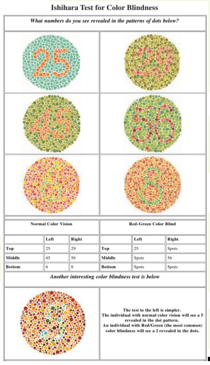

इशिहार चार्ट: रंग अंधत्व चाचणी करण्या साठी वापरला जातो

रक्तदाब तक्ता

BLOOD PRESSURE CATEGORY	SYSTOLIC mm Hg (upper number)		DIASTOLIC mm Hg (lower number)
NORMAL	LESS THAN 120	and	LESS THAN 80
ELEVATED	120 – 129	and	LESS THAN 80
HIGH BLOOD PRESSURE (HYPERTENSION) STAGE 1	130 – 139	or	80 – 89
HIGH BLOOD PRESSURE (HYPERTENSION) STAGE 2	140 OR HIGHER	or	90 OR HIGHER
HYPERTENSIVE CRISIS (consult your doctor immediately)	HIGHER THAN 180	and/or	HIGHER THAN 120

Figure 3: Sakhi Field Kit (Marathi) (Page 2/3)

NEEM
BY DVARA HEALTH FINANCE

Mann Deshi Foundation

काळजी पूर्वक संवाद कसा साधायचा

लक्षात ठेवण्याचे नियम

- "हा मधुमेही रुग्ण" असे कधीही म्हणू नका.
- तुम्ही म्हणू शकता, “या व्यक्ती ला मधुमेह आहे”.
- “मानसिक रुग्ण असे म्हणू नका”.
- तुम्ही म्हणू शकता, "मानसिक आरोग्य स्थिती असलेली व्यक्ती”
- "चांगले" किंवा "वाईट" सारखे शब्द देखील टाळा.
- त्याऐवजी, "उच्च" किंवा "निम्न" वापरा. किंवा अधिक चांगले, "सामान्य श्रेणीमध्ये" किंवा "सामान्य श्रेणीच्या बाहेर”.
- "रोग" किंवा "समस्या" शब्द वापरू नका.
- त्याऐवजी, "समस्या" किंवा "परिस्थिती" हा शब्द वापरा.
- म्हणू नका, कोविड ची लागण झाली आहे”
- म्हणा, "कोविड नी पकडला"
- असे म्हणू नका, "औषध सेवन करणारे".
- तुम्ही म्हणू शकता, “कधीकधी पदार्थ वापरतात”.
- जर एखादा रुग्ण तुमच्या सूचनांचे पालन करत नसेल, तर त्यांना सांगू नका की "त्यांनी ऐकले नाही तर त्यांची स्थिती बिघडेल".
- त्याऐवजी, "तुम्ही सुधारणा साध्य करण्यासाठी या सूचनांचे पालन करणे महत्त्वाचे आहे" असे म्हणा.

Figure 4: Sakhi Field Kit (Marathi) (Page 3/3)

B Swasthya Swaraj

B.1 Introduction

Swasthya Swaraj is a not-for-profit organization working towards making health a reality for the poorest and unreached. Swasthya Swaraj has set up a model community health programme – Swasthya Swaraj Comprehensive Community Health Programme in the tribal-dominated Thuamul Rampur block of the Kalahandi district of Odisha. Its program is active in 79 villages from 10 panchayats – Kerpai, Nakrundi, Kaniguma, Gunpur, Kiapadar, Thuamul, Sagada, Silet and Talaampadar and covers a population of about 14,000 people. However, people from six other blocks of Kalahandi and Rayagada districts also avail its services.

B.2 The *Swasthya Saathis* Program

In an area with poor transportation and communication facilities, with people deprived of healthcare for all these years, death rates are high. It is also difficult to find literate women in the community. As a result, in the 79 villages and hamlets (the intervention areas of Swasthya Swaraj), 86% of the recruited women are illiterate. All are tribals and are called *Swasthya Sathis*. The villagers selected them based on the criteria proposed by Swasthya Swaraj, and they are being trained once a month regularly. They bring with them deep cultural connections with their community, which ensures their effectiveness.

They're trained on the following topics and skills:

1. Topics for training:
   - (a) Duties and responsibilities of *Swasthya Sathis*
   - (b) Data documentation of vital events
   - (c) Community mobilization
   - (d) Communicable diseases: Malaria, TB, Diarrhoeal diseases & dehydration, ARI, Scabies
   - (e) Antenatal care, risk assessment of pregnancies, Intranatal care, postnatal care
   - (f) Newborn care
   - (g) Diseases of under-five children
   - (h) Nutrition, Growth monitoring, detection of Severe Acute-Malnutrition (SAM) & Moderate Acute-Malnutrition (MAM), community-based management
   - (i) Immunization
   - (j) First Aid
   - (k) Adolescent health and nutrition, their empowerment
2. Skills:
   - (a) Health communication skills

(b) Data documentation skills, disease surveillance

(c) Follow up of high-risk pregnancies at home,

(d) Conducting home delivery (in low-risk cases)

(e) Care of the newborn, weighing the baby,

(f) Diagnostic skills in diagnosing common infectious diseases and managing

(g) Checking temperature, BP, respiration rate, fetal heart rate

(h) Curative skills: correction of dehydration; age-adjusted doses of Paracetamol, Amoxicillin, and Chloroquine; treatment of scabies.

B.3 Community Health Practitioners (CHPs)

B.3.1 The problem

Tribal areas where indigenous people live are the most neglected parts and have the worst health and development indices in India. Odisha state has a tribal population of 22% consisting of 63 various tribes. 13% of these groups have been classified as particularly vulnerable tribal groups (PVTG). In these groups, the death rates are high, maternal mortality and morbidity are unimaginably high, there is high infant and under-five mortality, and infectious diseases like malaria and diarrhoea are present in epidemic proportions. Accessibility to healthcare is an important determinant in promoting health in tribal areas. A sick person cannot walk more than six kilometres to access healthcare, and when a mother is carrying a sick child, this distance may be reduced to three kilometres. The poverty of the tribals is so shocking that they cannot afford even low-cost healthcare services. Even though government uses various means, both attractive and coercive, doctors are not willing to work and stay in rural areas as long as the yawning gap in the development between rural and urban areas remains. And the problems in rural areas are far too complex to be managed by a village health worker (the *Swasthya Sathi*), ASHA (1), or ANM (2), at the level of training that they currently have, no matter how determined and motivated she may be.

Inadequacy, inequitable distribution, and lack of competence of the health workforce are constant problems in rural areas and, more so, in tribal areas. A critical look at today's nursing education points to one thing - nursing education though it spends many hours of teaching and exposure to community health and public health issues, is focused on producing a workforce for working in hospital-based healthcare facilities. The ANMs who are meant to work in the community have very limited knowledge focusing only on maternal and newborn health and immunizations through the sub-centre.

B.3.2 The proposed solution

Solutions for the vexing problems in tribal health issues have to be sought within the tribal community itself. Tribal girls who are educated, if empowered with knowledge, skills and competencies, and, above all, the motivation to work for the poorest and most disadvantaged in a professional way in hard-to-reach locations, a lot can be changed.

Tribal health issues have to be addressed in innovative ways to understand their health needs and problems, and life situations. The *One-size-fits-all* approach will not work in solving tribal health issues. Technological solutions have limited value in solving their problems in the initial stages. The bulk of diseases in tribal areas are constituted by communicable diseases and malnutrition, which can be tackled if there is a dedicated, competent workforce closer to people who can involve and support all various categories of health personnel and community. There is a need to look beyond the conventional healthcare workforce. The existing health system fails to provide the much-needed primary healthcare in the hard-to-reach tribal areas. As long as the urban-rural divide remains and keeps widening, the great disparity in the distribution of doctors and health professionals is sure to continue. In order to address this great disparity in healthcare services and the distribution of healthcare professionals in backward areas, there is a great need to search for and find alternative solutions other than conventional ones.

In tribal areas, the focus of *human resource in health* training needs to shift from the doctor-based strategy to "where there is no doctor" (3) - supporting a middle-level cadre of health professionals. The village-level health worker could continue to provide first-contact care at the village level, counsel for proper and timely referral, be a link person and also prevent illnesses and organise people to demand health care services from the government. But the new middle-level health professionals would be providing much-needed emergency care for common health problems, doing basic investigations, running chronic illnesses or disease-specific programmes with guidance from the physicians, investigating epidemics and following up on public health problems at the village level. Presently no middle-level cadres in the public health system are trained in the management of illnesses and the other above-mentioned functions in this iniquitous healthcare situation. Ayushman Bharat (4) has planned that B.Sc nurses with an additional six months of training and a Certificate in Community Health for Nurses (5) will be posted in Health & Wellness centres (4). But where doctors do not go, B.Sc or other nurses also will not go, more so in tribal areas.

B.3.3 Training needs of the middle-level health workforce

1. Diagnose common illnesses, including emergencies such as fractures, acute gastroenteritis, severe pneumonia, seizures, injuries, animal bites, acute abdomen, and severe malaria.

2. Manage these common emergencies and decide about the referral of those that she cannot manage.

3. Manage a basic laboratory and be competent in performing investigations of common public health problems such as tuberculosis, malaria, sepsis, urinary tract infections, and diagnosing pregnancy.

4. Investigate reported outbreaks of illnesses in the area served and plan its basic management.

5. Plan community control of common public health problems such as malaria.

6. Manage chronic illnesses such as hypertension, diabetes, sickle cell anaemia, rheumatic heart diseases, and chronic obstructive airway diseases after a physician has made the therapeutic plan at the community health centre.

7. Women and child care programme: running an augmented antenatal care programme, conducting home delivery/ institutional delivery, managing obstetric emergencies, care of the newborn, running an under 3-year-old child care programme.

B.3.4 Strategy: Diploma in Community Health Practice (DCHP)

As the *Swasthya Sathis* cannot be trained beyond a limit, Swasthya Swaraj has started another cadre of tribal health workforce - middle-level health workers referred to as Community Health Practitioners (CHPs). These are class 12 passed tribal girls who are trained through a two-year full-time diploma course affiliated with a university (the Centurion University in Odisha). This course, the Diploma in Community Health Practice (DCHP), designed by Swasthya Swaraj, combines public health knowledge and skills (sociology, social anthropology, basic epidemiology, etc.) with clinical skills and knowledge. They are, in fact, excellent community nurses with good community mobilization skills and clinical skills - ideally suitable for the tribal areas.

The DCPH, a two-year Diploma programme for disadvantaged tribal girls conducted in the tribal context of the villages of the Thuamul Rampur block in the Kalahandi district of Odisha, in collaboration with Centurion University in Bhubaneswar, is a step towards solving the problem of high disease load and deaths along with poor access and availability of healthcare in tribal areas. The aim of this course is to prepare a cadre of mid-level health workers who are selected from disadvantaged communities (tribal) who are trained well with sound public health knowledge, clinical skills and competencies, and communication skills in preventive, promotive and curative aspects of health to care for the health needs of the population in hard to reach tribal pockets and areas. The training is done largely in the tribal setting by competent doctors, public health professionals and nurse educators. After the training, they will be staying in inaccessible areas and delivering comprehensive primary healthcare as full-time staff of the Comprehensive Community Health Programme.

The DCPH is a 2-year full-time course with a 6 months compulsory internship in remote tribal villages. Students who graduate from this course are called Community Health Practitioners (CHPs). CHPs will be an integral part of community health programmes - providing quality primary healthcare, running health & wellness centres, maternal & newborn health services, comprehensive obstetric care, immunization services, health education & communication, community mobilization, care of the mentally ill, elderly & disabled in the village, etc. in the hard to reach tribal pockets – in areas “where there is no doctor” (3).

After completion of studies and obtaining the Diploma, the graduates are required to work in remote tribal areas for a minimum of two years. After the completion of the two-year service period during which they will be paid full salary, they may or may not continue in the area and may move into a hospital setting; those who want to pursue higher studies in public health or other professional courses in health will be guided and helped accordingly. The course is designed in such a way that it prepares the students to serve the disadvantaged with competence as well as encourages the students to acquire higher professional qualifications

in health sciences or public health.

1. Brief about DCHP Programme:

    (a) Duration: Two-year full-time, residential course affiliated with Centurion University in Bhubaneswar (CUTM)

    (b) Venue: School of Community Health Science & Practice, Kaniguma village, Thuamul Rampur Block, Kalahandi district, Odisha.

    (c) Maximum number of seats per year: 10

    (d) Course curriculum is designed and tailored to the tribal context and to address health needs.

    (e) Girls coming from tribal areas of different districts or states have to work in the tribal areas they come from upon completion of the course.

    (f) Course is completely free of cost with an understanding to work for a minimum of two years in tribal areas.

    (g) Competent doctors and nurses will take the classes, and public health specialists who are experienced in working at the grassroots.

2. Selection criteria:

    (a) Minimum educational qualification: Class 12 passed (preferably Science group)

    (b) Socioeconomic background: Girls who have completed 18 years of age; Belong to Tribal /ST families (Adivasi); Economically backward families

    (c) Non-negotiable criteria: Girls who are willing to live and work in underserved Adivasi areas; Consent from family members

B.3.5 The need for the DCPH

The perennial problem in rural areas and more so in tribal areas is the acute shortage of healthcare human resource and their unwillingness to work in these areas. Swasthya Swaraj trains a cadre of Swasthya Sathis. The Swasthya Sathis are the women selected by the people – one woman per village and per hamlet. 92% of these women are illiterate as the female literacy levels in tribal pockets are abysmally low. These women are motivated and reach out to people in their own villages, and they are the key contact person in each village and hamlet. But there is a limit to which they can be trained as theirs is a part-time job, almost like that of a volunteer, and it has proven difficult for the people to have faith in her competence.

Formally trained nurses (General Nursing and Midwifery, B.Sc Nursing) with the registration of the Nursing Council are trained by the government and private institutions for working in well-established hospitals. The ANMs (Auxiliary nurse midwives), who, too, are trained by the government and private institutions and are registered with the Nursing Council, are expected to be in the community, running sub-centres (one sub-centre for 5000 population). But their training and skills are limited to only maternal and newborn health and immunization. In tribal areas, this cadre is largely found to be a failure mainly because

of the difficult terrain and distance, scattered villages and culture of the people, which a nontribal woman from outside finds difficult to manage.

There is a great need for mid-level health workers to fill the gap and provide quality healthcare to the community where different categories of people have to be reached out to and managed - pregnant women for antenatal care, conducting delivery, postnatal care, newborn care, under-five children, school going children, adolescent girls and boys, youth, elderly, disabled, destitute and orphans, chronic diseases, acute infectious diseases, non-communicable diseases, etc. Many hospitals run informal training programs for mid-level health workers who can only be appointed in the hospitals as nurse assistants/nursing aids and have no formal qualifications.

Inadequacy, inequitable distribution, and lack of competence of the health workforce are constant problems in rural areas and, more so, in tribal areas. In spite of all these problems, primary healthcare has to be made available in the areas where the poor live, and it is a question of saving many young tender lives and the lives of mothers. The bulk of diseases in tribal areas are constituted by communicable diseases and malnutrition, which can be tackled if there is a dedicated, competent workforce closer to people who can involve and support all various categories of people in the community.

A critical look at today's nursing education points to one thing - nursing education though it spends many hours of teaching and exposure to community health and public health issues, is focused on producing a workforce for institution-based healthcare facilities. Community health nursing is visualized by Swasthya Swaraj to produce a cadre of competent health workforce who are trained in the community and will practice in the community. Curative care, disease prevention, health promotion, working with various groups of people in the community, making the CBOs work, and supporting and capacity building of all grass root level health workers will be carried out by this cadre. This will be a competent workforce capable of implementing all national programs at the grass root level, producing innovation, knowledge, and evidence-based practice of medicine in the community. The community nurse has a far greater responsibility than the average doctor or nurse. The doctor feels a responsibility for those sick or injured persons who come to him/her- those whom he/she sees as patients. But the Community Health Nurse (CHN) or CHP is responsible to the entire village or communities of people where she lives and works. Her concern is for the health and well-being of all the people. She does not wait for those in greatest need to come to her. She finds out who they are and goes to them.

This course designed by Swasthya Swaraj and Centurion University formalizes this training, and they get a diploma which gives them opportunities to further go ahead with studies of graduation and post-graduation in related subjects. This is a step in empowering tribal women with leadership. In remote tribal areas, the need is not for big hospitals with all the technology-based facilities but for trained human resources who will go to the hard-to-reach areas and deliver healthcare and promote health. Small centres with facilities for emergency care will do better than big hospitals located far away from people. Swasthya Swaraj is trying to train and empower tribal women to care for their own health, which is the only sustainable solution.

For regular practical learning, Swasthya Swaraj health centres and project villages are used. But for specific postings, the students are posted in private, non-profit, and government healthcare facilities. The course is completely free of cost for the students, including their

boarding & lodging. The students also receive a small monthly stipend. Swasthya Swaraj raises funds to meet all the expenses.

B.3.6 Duties of the CHP

1. Running the *Gaon Swasthya Poshana Gharo* or GSPG (Village Health & Nutrition Centres)
2. Curative services: communicable and non-communicable diseases.
3. Management of medical emergencies, suturing, abscess draining, etc.
4. Running a basic rural laboratory which will help in arriving at diagnoses.
5. 100% antenatal coverage, postnatal coverage and home-based newborn care.
6. Conducting deliveries in the GSPG or at home (low-risk deliveries)
7. Assisting in immunization and achieving ¿90% coverage in her villages
8. Training of adolescent girls, mothers, and school children in health & nutrition related subjects
9. Conducting weekly or bi-weekly clinics with the help of a doctor and health team from Swasthya Swaraj health centre.
10. Growth monitoring of all under-five children, early detection of nutritionally at-risk children
11. Management of severe acute malnutrition in the GSPG and in the community
12. Care of the elderly and disabled in the community.
13. Referral of cases to Swasthya Swaraj health centres or to nearby government healthcare facilities.
14. Proper running of community-based organizations like Gaon Kalyan Samitis, school management committees, and village health planning committees.
15. Ongoing training and handholding support to village health volunteers (*Swasthya Sathis*, ASHAs).

B.3.7 Uniqueness of the CHP

1. Many healthcare institutions and community health projects have been training women as nurse assistants or health auxiliaries, or senior health workers. But none of these courses was mainstreamed as the Nursing Council of India was always opposed to them and was sceptical about the quality of these programs. These students could not go further from the level they were trained.

2. This is the first time a mid-level health worker course (other than the conventional nurses and ANMs) combining public health knowledge and clinical expertise has been mainstreamed by affiliating it with a State University.

3. These women, after completion of their rural service, if they wish to go for higher studies in public health or other affiliated health subjects, will be able to go for it and will be encouraged to do it so that they are able to contribute to nation-building effectively.

4. This tribal cadre is much better equipped, relative to village health workers like ASHAs and ANMs, with knowledge and practice of public health and clinical skills and knowledge, teaching capability and leadership skills, and community mobilization skills.

B.3.8 Scalability of DCHP

This project was proposed after some years of experience working in the tribal areas and understanding the depth of the problem after doing a pilot project for two years. Across India, the problem in remote tribal areas is the same, with some variations in magnitude. These are areas where it is extremely difficult to get doctors and retain them and also nurses and paramedics. But these tribal areas have a high burden of preventable and easily treatable diseases and preventable deaths. This well-trained tribal cadre is ideally suitable to take care of the healthcare needs of the tribal and other similar remote rural populations.

B.3.9 Case Study of CHP Anjana

Anjana is from Pustiguda village in the Thuamul Rampur block of the Kalahandi district of Odisha. She is the eldest child of a single-parent family. Her family is poor with her father being a marginal farmer. Anjana was always passionate about serving the community, and that's why she got admission to an ANM course after she finished high school. But due to financial problems, she had to drop out of that course. She then joined Swasthya Swaraj as an outpatient support staff member and soon thereafter was enrolled in the Diploma in Community Health Practice (DCHP) course.

She was enrolled in the DCHP course in the first batch in 2018 and completed the course in 2020. During the course, she got experience in conducting more than ten deliveries at Saheed Hospital as a part of their practical studies. After completing the course, she was posted at the remote health centre at Silet village in the Kalahandi district of Odisha and, after six months there, was transferred to Swasthya Swaraj's Kerpai Health Centre located in the same district, and has since been serving the community there. Soon after her posting there, for example, she was able to successfully deliver a baby in a complicated situation at the Centre, even though she happened to be the only person on duty at that time. With the telephonic guidance of one of the Swasthya Swaraj physicians, she was also able to address the retained placenta complication that arose after the baby was delivered, even though it was the first time she had handled such a complication.

C Ambuja Cement Foundation

C.1 Introduction

Ambuja Cement Foundation (6) is an independent development organisation with a presence across twelve states in India and works towards enhancing overall prosperity in all the rural communities it serves. They have two long-standing (over 20 years of history) community health worker programs – one referred to as the *Swasthya Sakhi* (human-health friend) and the other as the *Pashu Sakhi* (animal-health friend). In both of these programs, health workers are hired locally, assigned to a defined population, trained broadly in medical matters, and required to offer comprehensive medical services to that defined population. This note discusses in some detail both these programs.

C.2 The *Swasthya Sakhi* Program

Their work on human health had a modest beginning in 2001 when ACF realized that the lack of facilities at the primary health outposts was the prime reason for high mortality among mothers and children. In 2002 ACF started providing health services at the village level through a mobile dispensary. But it soon realized that women did not avail of these services. ACF then decided to train women at the village level to provide medical services. They were originally called Village Health Functionaries (VHFs) and were later renamed as *Swasthya Sakhis*. To address the issues and problems, after much research, brainstorming and assessment of the options available, ACF decided to work on the task of creating a cadre of *Swasthya Sakhis* that would be drawn from the community. The model of *Swasthya Sakhi* was based on the concept of "barefoot doctors" that has been tried and tested over ages, especially in rural China (7). The requirement for *Swasthya Sakhi* was to have the basic diagnostic and first aid skills, the ability to do primary clinical examinations and administer over-the-counter medicines to attend to the common illness of their own villagers. They were also expected to be able to create awareness about public health and hygiene and initiate community participation in preventive healthcare.

ACF partnered with the Foundation for Research in Community Health (FRCH), a Pune-based non-profit focused on primary care, to create a training model suited for these very tasks of skill creation. *Swasthya Sakhis* were selected from villages with the underlying assumption that the person was local, so available immediately; being from the village, the person understood and knew the local health problems that are endemic to the area or, if there is an outbreak, they would be easily reachable. Also, the villagers had less difficulty in establishing rapport with someone from their own community. FRCHs principal role was to conduct the training as well as monitor the progress of the trainees at regular intervals. ACF took up the responsibility of funding the project, selecting the *Swasthya Sakhis*, and carrying out the ongoing monitoring and evaluation of their work.

C.2.1 Role of *Swasthya Sakhis*

The *Swasthya Sakhis* initially began with a curative approach to meet the immediate pressing clinical needs of the community, and over a period of time, also started providing services

on preventive and promotive health services to the community.

1. Curative services provided by the *Swasthya Sakhis* included attending Community Clinics and assistance in health camps. Provision of primary healthcare services at the village level, referral and follow-up of patients.

2. Preventive services by *Swasthya Sakhis* included need/trend assessment for illness in the village.

3. Awareness sessions on identified health issues.

4. Home visits to promote preventive measures at the household level.

5. Health education for ANC/lactating mothers, anaemic cases, adolescent girls, organizing village meetings as well as supporting ACF in organizing them, record keeping.

6. The promotive services provided by *Swasthya Sakhis* included strengthening Village level Institutions like local governments and community-based organisations by attending their meetings and raising issues and linkages with the government village-level health system and with various government schemes.

After the initiation of this programme, the community started accepting *Swasthya Sakhis* as their own and sharing their problems with them for their advice. They were of the opinion that the *Swasthya Sakhis* were of help immediately when the need arose; they were very friendly, helpful and capable of proper diagnosis. They also said that having access to health services in the village saved their time and energy. This project was found to be monetarily beneficial as villagers saved their time as well as money in travelling to get medical attention and on the cost of the service.

C.2.2 ***Swasthya Sakhi*** selection & training

1. *Swasthya Sakhi* selection criteria:

   (a) Minimum educational qualification should be 8th pass.

   (b) Should be keen to do this work.

   (c) Should have good communication skills.

   (d) Should be familiar with the culture and geographical setting of the villages.

   (e) Should have leadership qualities and good rapport with village Panchayat, Sarpanch, etc.

2. *Swasthya Sakhis* are trained in the following aspects:

   (a) Identification of high-risk pregnant women and high-risk neonatal care and the importance of 1,000 days.

   (b) Awareness of the topics covered for adolescent health.

(c) Counseling among eligible couples for family planning and use of reversible methods.

(d) Identification of malnourished children and precautions to prevent malnutrition.

(e) Identification of TB patients, cause, symptoms and treatment for prevention

(f) Screening of Hypertensive and Diabetic patients, generating awareness on cause and symptoms of Diabetes and Hypertension.

(g) Symptoms and effects of tobacco consumption in first-trimester pregnant women and the harmful effects of tobacco.

(h) Training on Mental Health and identification of Common Mental Disorders (CMDs) and Severe Mental Disorders (SMDs), counselling sessions and facilitation of referral services.

(i) Training on detection of early signs and symptoms of cervical, breast and oral cancer.

(j) Training on telemedicine and organising teleconsultations.

C.2.3 Interventions by *Swasthya Sakhi*

1. Maternal and child health-related interventions:

   (a) Conducting awareness on Menstrual Hygiene Management among adolescents, distribution of sanitary napkins, overseeing the installation of sanitary vending machines and incinerators at schools, and educating young people about these issues using learning materials and models developed by experts.

   (b) Strengthen ante-natal and postnatal service, home-based neonatal care, and immunization adherence with a particular focus on high-risk antenatal and postnatal cases.

   (c) Early identification of pregnant women by monthly visits to eligible women. Addressing anaemia and building overall awareness of issues related to nutrition in them through counselling and demonstrations. Facilitation of monthly checkups and nutritional supplements like iron and folic acid (IFA) and calcium tablets, and tetanus injections for these women. Nutrition and other counselling every trimester. Helping women avail government-provided antenatal services and, if required, accompanying them to government centres.

   (d) Helping women prepare for their delivery to ensure they have the best possible experience during labour.

   (e) Promotion of reversible or permanent family planning methods amongst eligible couples through counselling and other methods.

   (f) Provide information about the importance, duration, and frequency of breastfeeding to lactating mothers.

   (g) Supporting the local government run *Anganwadi* (day-care) centres in multiple ways, including in the area of proper measurement of her height, weight, and MUAC (mid-upper-arm circumference) for growth monitoring of the baby.

2. Communicable disease-related health interventions:
   (a) Focus on referrals and follow-ups and the nutrition needs of patients.
   (b) Particular focus on tuberculosis patients.
   (c) Support government efforts relating to tobacco cessation amongst pregnant mothers along with counselling and awareness generation on the harmful effects of tobacco amongst adolescents.
3. Non-communicable disease-related interventions:
   (a) Assessment of NCDs through home visits using the CBAC assessment checklist (8).
   (b) Identification of high-risk patients through these assessments and their monthly follow-ups.
   (c) One-to-one and one-to-many Health awareness sessions
   (d) Community Mobilization for screening camps
   (e) Facilitating access to more advanced referrals, diagnostics, and treatment to ensure a continuum of care.
   (f) Create Support groups

C.2.4 *Swasthya Sakhi* Community Clinics

This is a variant of the *Swasthya Sakhi* program in which for each community of about 1,000 individuals, there is a fixed clinic that is set up with, among other things, a sphygmomanometer, a spirometer, a thermometer, and a pulse-oximeter. It also has a full complement of medicines available at the facility. In this clinic, all of the work is done by the *Sakhi* assigned to the community, but at the moment, there is also a doctor who visits once a week, and that is the only day on which medicines are handed out. There are, at the moment, no treatment protocols that are followed by either the *Swasthya Sakhi* or the doctor. All of the services offered by the clinic are on a fee-for-service basis, and the fees are collected by the Village Development Committee, which also owns the clinic. At the moment, nine of these clinics are being piloted in the Chandrapur district of Maharashtra by ACF.

C.2.5 *Swasthya Sakhi* stories from Chandrapur, Maharashtra

The work initially started in Chandrapur in 12 villages falling in the core area of the Ambuja cement plant with 21 *Swasthya Sakhis* but quickly expanded to cover 160 villages through 180 *Swasthya Sakhis* across three blocks of Chandrapur district, namely, Rajura, Korpana and Jiwati. Through its *Swasthya Sakhis*, ACF reached out to 16,312 households covering a population of over 85,000 individuals in the year 2013.

At present, in the Chandrapur district, the ACF health program operates in 70 villages across 3 blocks in Chandrapur district, namely Korpana, Rajura and Jiwati. The focus of the program is on providing home-based neonatal care where required and offering comprehensive healthcare services to all the covered households, with a network of 67 *Swasthya Sakhis*

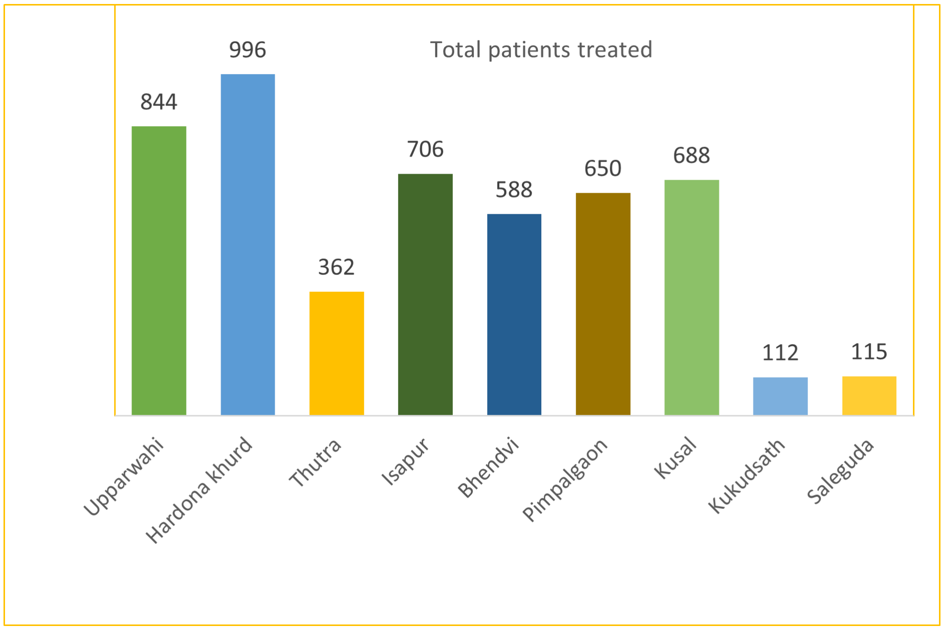

Figure 5: Community Clinics: Numbers of patients treated (Oct 2019 to Dec 2022)

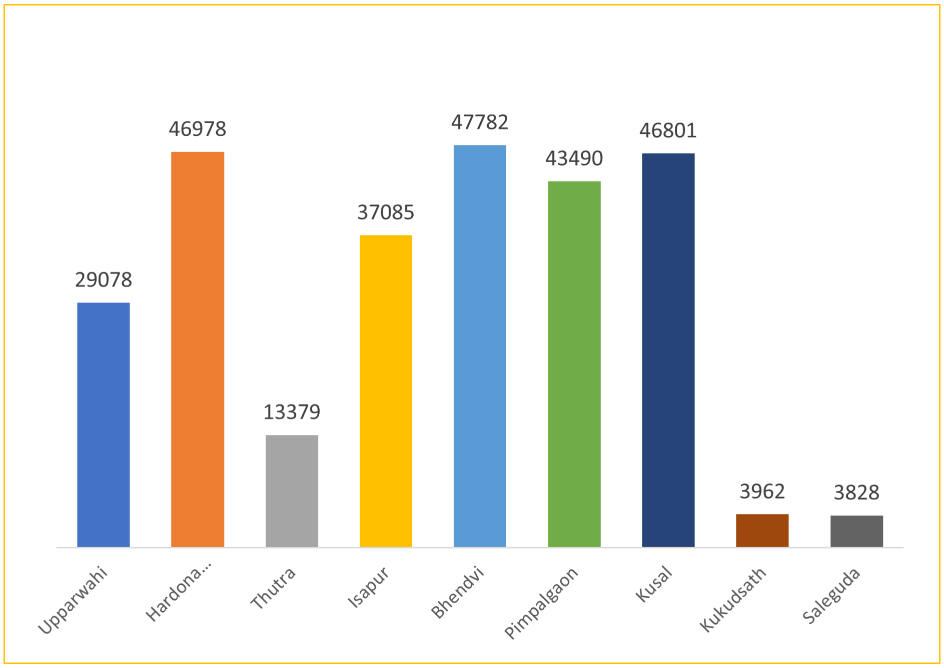

Figure 6: Community Clinics: Cumulative surplus in rupees (Oct 2019 to Dec 2022)

reaching 9,077 households and serving a population of 41,502 members. In 2023 4 more villages have been added to the program. The services provided by the *Swasthya Sakhis* under the program include the following:

1. Comprehensive curative services through nine Community Clinics.
2. Maternal and Child Health.
3. Tobacco cessation counselling.
4. Promotion of overall adolescent health (nutrition, sexual & reproductive health, mental health, prevention of injuries & violence, prevention of substance abuse) through a peer-led, life skills-based, behaviour change programme called APEKSHA (Adolescent Peer for Enhancement of Knowledge, Skill and Healthy Association).
5. Sanitation and Hygiene
6. Non-Communicable & Communicable Disease
7. Addressing mental health issues with the *Sakhis* acting as lay counsellors using the *Atmiyata* approach pioneered by the Centre for Mental Health Law & Policy at Pune (9).
8. Menstrual Hygiene Management
9. COVID-19 Disease Awareness
10. Malnutrition

C.2.6 *Swasthya Sakhi* Program Achievements

1. A number of indicators relating to maternal and child health have shown sharp improvements within the area covered by the program. These include the stillbirth rate, neonatal mortality rate, infant mortality rate, and child mortality rate. The *Swasthya Sakhis* have played an important role in this through the information and counselling they provided and early identification and referral to specialists of high-risk pregnancies. They also helped alter some of the tribal practices, such as that of completely unassisted childbirth.
2. Inspired by the governments' "Swacch Bharat Mission" (10), recognising open-defaecation as an important social determinant of health, the ACF health program has been working towards making the villages open-defaecation free (ODF). In one of the villages, Baleguda, the fact that the *Swasthya Sakhi* was a member of the local community helped make the village 100% ODF because she was able to persuade one of the holdouts by tying a *Rakhi* (11) to him and asserting the customary rights of a sister that tying the *Rakhi* gave her.

3. In the remote block of Farakka in West Bengal, where the availability of government health services was patchy, the *Swasthya Sakhis* worked with pregnant women to help them determine their Expected Delivery Date (EDD) by, among other things, ensuring the availability of pregnancy-test kits so that they could move to a location near a functional government hospital in good time and also be otherwise better prepared to deliver their baby. Having determined if a woman is pregnant, they were able to provide these women with iron and folic acid (IFA) and calcium tablets, measure their blood pressure and their haemoglobin levels, and also make arrangements for them to get an ultrasound done. For example, Money Hembram, the 24-year-old wife of Salku Soren of Kalidanga village, reported that "my menstrual period typically starts on the 13th of every month, but in 2021 I did not start my period even after the 25th of January. I told this to *Sakhi* Didi [the local *Swasthya Sakhi*], and she ask me to do a pregnancy test. I was worried about where to go and how to test. *Sakhi* Didi said don't worry and took care of it. And through this test, I came to know that I am going to be a mother". During 2022-23, the *Swasthya Sakhis* identified 1,303 women of reproductive age. They also provided 992 couples with reversible methods of contraception and 864 women with Iron and Folic Acid (IFA) tablets. Due to efforts of the *Swasthya Sakhis*, the proportion of women below the age of 20 who are pregnant has also declined from a high of 39.77% to 13.20%.

4. In Chhattisgarh, 27 *Swasthya Sakhis* are active in 13 villages, catering to a population of 26,120 people. Each *Swasthya Sakhi* serves, on average, 968 people. in the villages where the *Swasthya Sakhis* are present, women delivering in hospitals has increased from a 90.56% in 2018-19 to 96.03% in 2022-23 (figure 7), the mortality rates have also fallen sharply (figure 8), and the immunization rate has increased from 64% to 100%. 616 women were provided antenatal and postnatal support for early registration of their pregnancies, timely vaccinations, for taking care of premature and underweight babies, and measurement and treatment of blood pressure and anaemia. In order to address the increasing burden of non-communicable diseases (NCDs) in the area, 4,547 people were screened at 44 camps, and 4,273 people were given advice on lifestyle management (LSM) at 324 awareness camps. Under the APEKSHA program (discussed earlier), 1,413 people participated in 173 *Ma-Beti Sammelans* (mother-daughter meets). In addition, multiple programs to address malnutrition, tobacco cessation, mental illness, and cancer screening have been organised.

   > Arti Sahu a 55-year-old woman from Rawan village, reports that "*Sakhi* Amreika Patel stood beside me during my toughest times. I had no idea about the bad impact of Diabetes. One day I noticed an injury on my foot. I didn't take it seriously as I thought it to be a simple wound which was to recover gradually. But the condition worsened, and within 15-20 days, I started having pus and pain and was not able to walk properly. I felt very weak, and seeing my condition, my family members were also worried. When Amreika Patel saw my wound, she suggested that I check my sugar level. My blood pressure was found to be 168/82 mmHg and sugar 490mg/dl. I followed her advice, and gradually, my foot also recovered, and finally, I

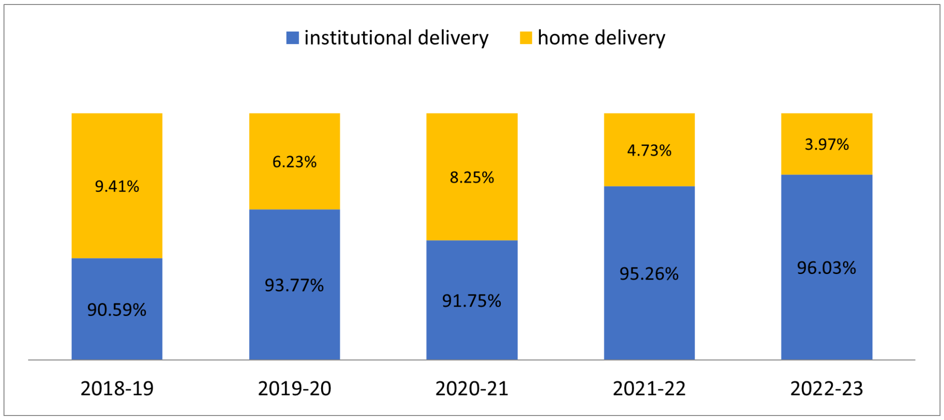

Figure 7: ACF Chhattisgarh Institutional Delivery Rates

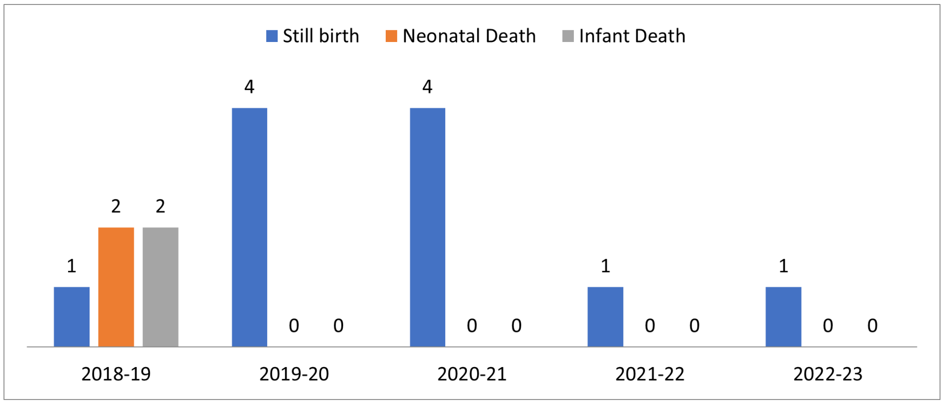

Figure 8: ACF Chhattisgarh Mortality Data

got relief from tension".

5. In Ambujanagar in the Junagarh district of Gujarat, the *Swasthya Sakhis* have ensured that 100% of the women deliver in hospitals and there is 100% immunization. This has helped in reducing the maternal mortality rate, the neonatal mortality rate, and in improving the hygiene practices of rural women and the adoption of good health practices by them.

C.3 ACF *Pashu Sakhi* Program

Pashu Sakhi (animal friend), like the *Swasthya Sakhi*, is a woman from the village trained to handle routine medical requirements for animals like goats and cows. While they are not trained to handle surgical interventions (though, when needed, they do assist the animals in the routine delivery of their kids and calves), they can identify diseases, provide medication and vaccinations, perform castrations, and suggest remedial or preventive measures. The goal of the program is to advise farmers about health check-ups for their livestock, vaccinations, deworming, hygiene, breeding, feeding, and the management of animal wastes. *Pashu Sakhi* receives both structured classroom training and field practice. The *Pashu Sakhi* is a goat

rearer herself who actively uses her skills to help her neighbour goat farmers within the village. Experiences of a number of grass root organizations indicate that a village-based knowledge extension service cum preventative health care service through training local small-livestock farming women is a proven and effective strategy to improve management and reduce mortality & morbidity on a significant scale. These women offer doorstep services like livestock vaccinations, de-worming, hygiene, improved breeding, and feeding like Mineral mixture, Azola, Mineral block, and Goat manure made from animal waste. This will enable last-mile coverage in rural areas where clinical services for livestock are not available on time or are too expensive for the rural poor to afford[1].

The *Pashu Sakhi* program is a partnership for income generation and livelihood support of women farmers and landless families.

1. Objectives:
    (a) Promotion of goatery as income generation and livelihood support activity among poor rural women.
    (b) Empowering women from self-help groups (SHGs) to take the lead in goat health improvement & productivity enhancement.
    (c) Provide doorstep knowledge and services to reduce the risk of goatery arising principally from mortality and morbidity of goats.
    (d) Empowering the economic and social status of rural women.
2. Program Focus areas:
    (a) Mobilization and selection of SHGs women for livestock business.
    (b) Establishing small & large scale goat, poultry & milch cattle businesses.
    (c) Carrying out regular deworming & vaccination of animals.
    (d) Holding regular animal health check-up camps.
    (e) Establishing self-training centre & goat service centre.
    (f) Establishing a breed improvement program.
    (g) Establhsing an Artificial Insemination centre.
    (h) Establhsing a fodder & feed management program.
    (i) Developing a master training program.
3. Key Features:
    (a) A three-level (introductory, practical, and advanced) 7-day training programme on how to take care of goats.

[1]ACF's *Pashu Sakhi* program is not the only one in India to adopt this model. The World Bank, for example, has worked with the Jharkhand government to develop a similar intervention (12; 13).

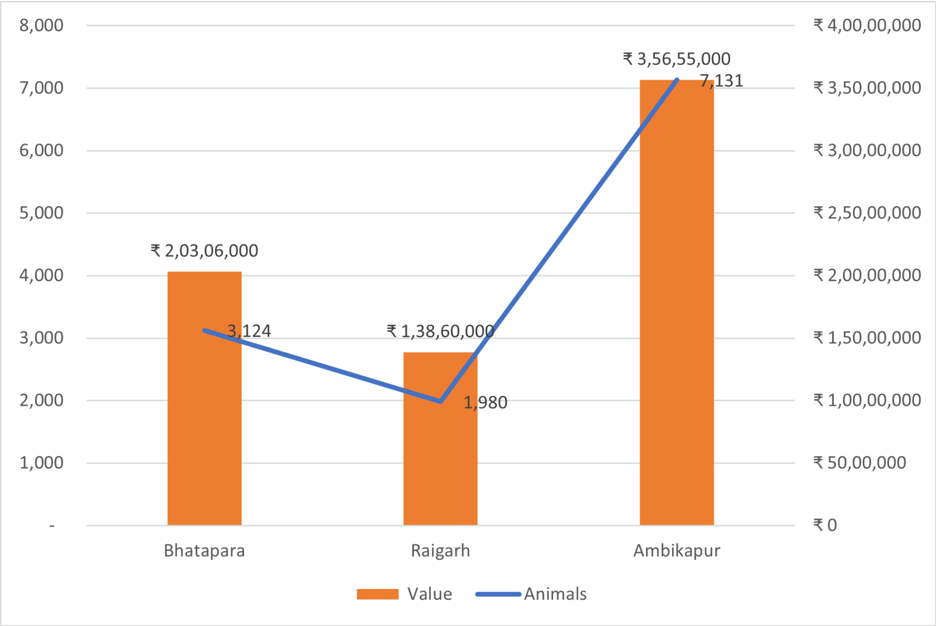

Figure 9: Herd Sizes and Value in Chhattisgarh Villages

(b) After the 7-day formal training, they are provided with technical expertise on taking care of livestock, advising fellow villagers on the economic benefits of rearing livestock for sale, and connecting farmers to Goat Based Livelihoods Groups (GBLGs) and traders, thus helping them get better access to markets to sell their produce.

(c) Acting as an interface between the Animal Husbandry & Veterinary Departments of state governments and goat farmers.

(d) Each *Pashu Sakhi* covers over 40-50 households.

4. Impact:

(a) Reduced the goat mortality rate.

(b) Increased the practice of regularly deworming & vaccinating goats.

(c) Increased the number of goat-rearing families.

(d) Improved the social & financial status of women goat farmers.

C.3.1 *Pashu Sakhi* Impact in Chhattisgarh

In Chhattisgarh, livestock production is in the hands of marginal and landless farmers, with women accounting for over 70% of the production. In fact, many of the *Pashu Sakhis* own livestock themselves. The *Pashu Sakhi* program of ACF covers 215 households in 27 villages. A total of 1,867 goat farmers are associated with the program, and they have a total of 12,235 animals between the ages of 4-18 months with a total value of about ₹7 crore (70 million). Figure 9 shows the distribution of the animals in the three clusters of villages.

Impact Area	Before	After
Goat mortality rate	5-10%	Reduced to 1%
Growth rate	Goat 9-10 kg<br>Buck 12-13 kg	Goat 12-13 kg<br>Buck 15-16 kg
Feeding Practices	Traditional (only open grazing)	Open grazing with stall feeding.
Improved Breed	Slow growing local breeds only.	Good quality bucks have been introduced from the outside for cross-breeding; these new bucks are also now being bred locally.
Goat Price	Goat: ₹3500 @ ₹350/Kg	Goat: ₹4550 @ ₹350/Kg
	Buck: ₹5850 @ ₹450/kg	Buck: ₹7200 @ ₹450/kg
Increased Income	₹13,000-25000/household / year	₹30,000-50,000 / household /year
Goat Manure	Throw in open field	Make goat manure and sell @ ₹20/Kg
Herd size	3-5 / household	6-11 / household
Healthcare costs	₹500 rupees / household / month	₹200-250 / household / month
Marketing	Earlier rate decided by butcher on estimated weight.	Now farmers are weighing before negotiation. The farmers decide the actual price.
Goat shed	Problems in housing goats, disease-burden high, and herd size small.	After installing sheds, the herd size has gone up, input costs have reduced, as have the thefts of goats.
Sustainability	No plan	Every location, i.e., Raigarh, Amikapur and Bhatapara, now has a Goat Service Provider Group (GSPG); *Pashu Sakhi* incomes have increased in all locations as a result.

Table 1: Impact of *Pashu Sakhi* Program in Chhatisgarh

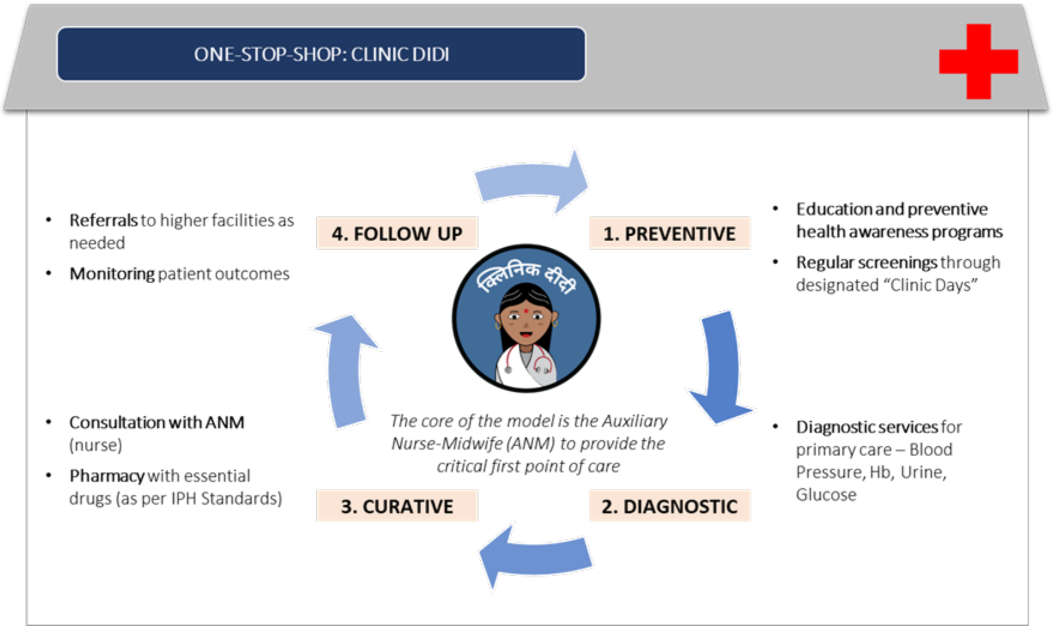

Figure 10: The Clinic Didi Model

D Clinic Didi

D.1 Introduction

The Clinic Didi (meaning *elder-sister-at-the-clinic*) model (figure 10) is a systems-based intervention. It is a primary care model at the first point of contact, designed to support an Auxiliary Nurse Midwife (ANM) to deliver a range of primary care services using technology under the supervision of a remotely located doctor. The ANMs are women employed by the government, receive 18 months of training, and are placed at either a Sub Health Centre (2) or a Primary Health Centre. The Sub Health Centre (SHC) is typically a village-level clinic intended to serve a population of 5,000 individuals, while a Primary Health Centre (PHC) is a small rural government hospital serving about 30,000 people (14). The ANMs are recruited through national or state-level selection processes and may not always be from the immediate area or local community that they are meant to serve. The program delivers primary care under a hub-and-spoke model. It was piloted across three SHCs and one PHC in the Jawhar block of the Palghar district of Maharashtra.

The key goal of the pilot was to ascertain the ability of the Clinic Didi program to provide all three parts of primary care, preventive, diagnostic, and curative, under one roof and provide effective follow-up and referral services to higher levels as needed (whether the hub at PHC or larger hospitals when needed). This outpatient clinic was set up in conjunction with regular ANM duties to run two hours a day, four days a week. The programme was seeded at the Institute for Transformative Technologies and was supported by multiple partners, including Dimagi and Sewa Rural.

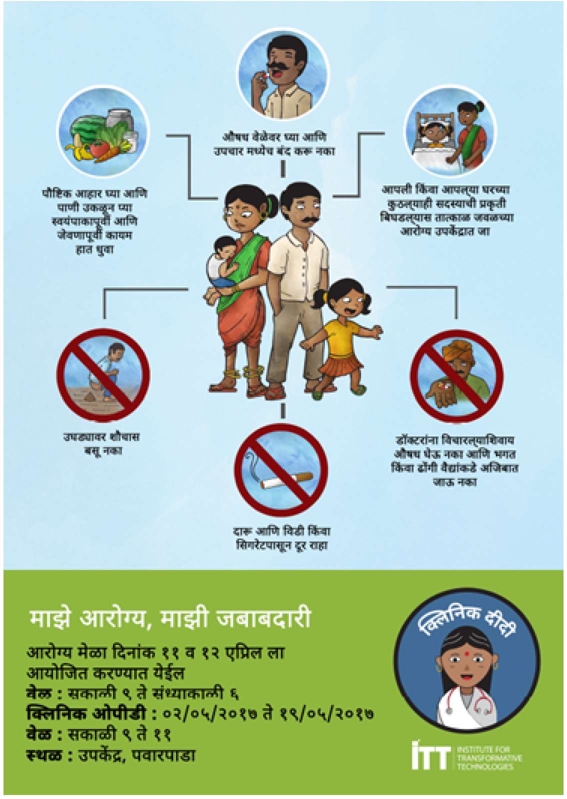

Figure 11: Clinic Didi Behaviour Change Poster (Marathi)

D.2 Components of the Clinic Didi Model

1. Effective technology for the SHC through a simple digital platform in the local language and tailored for a nurse-practitioner to record patient data and manage clinic supplies, as well as do basic vital tests using point-of-care diagnostic devices (Blood Pressure, Urine, Haemoglobin, Temperature, Pulse, Oxygen).

2. Targeted training, including protocol-based refresher training for ANMs to run outpatient clinics and diagnose and treat 21 basic primary care conditions (in accordance with their policy mandate at the time), targeted training for software and diagnostic devices, and training on clinic and patient management skills.

3. Patient & community engagement, on understanding the services available at SHCs, the importance of seeking timely care, challenges with faith-based or informal healers, and the importance of wellness and healthy habits. Figure 11 is an example of the kinds of local-language materials that were used to communicate the need for timely care, medication adherence, and healthy habits.

4. Improved operating processes on managing the ANMs, their schedule, and the clinic inventory infrastructure.

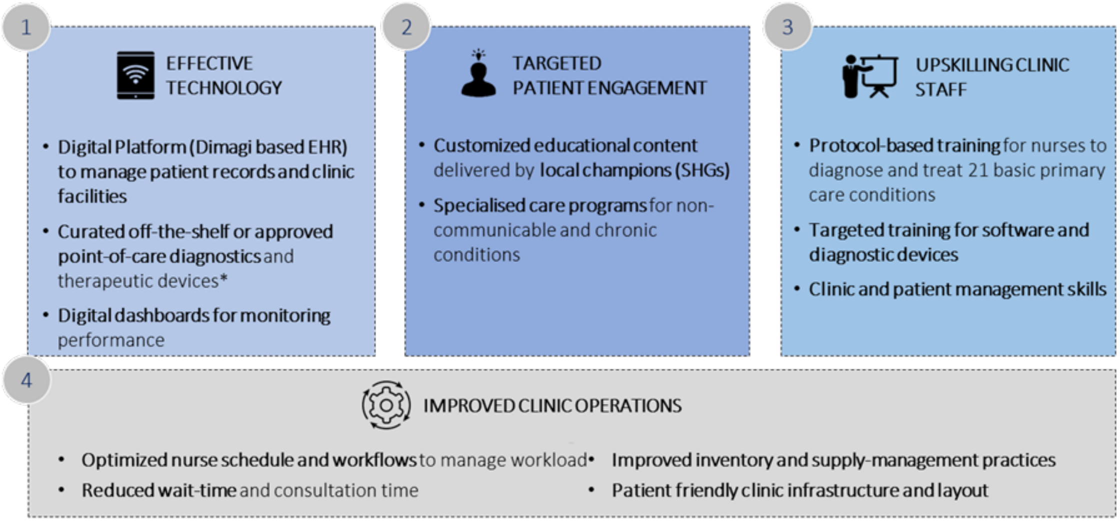

Figure 12: Components of the Clinic Didi Model

Healthcare interventions typically take years to show improvement in long-term health outcomes. In the short term, performance can only be measured through utilization, satisfaction, and quality of care demonstrated through improved processes and activities. These metrics can relate to the suitability of the intervention as well as the immediate uptake from patients and feedback on facilities. To measure the initial efficacy of the intervention, the performance of the clinics was measured on three parameters.

1. Utilization of the clinics, as defined by the numbers of patients registered and unique patient visits, to measure improvement in accessibility of primary care to patients.

2. Patient satisfaction scores to test if improved services at the SC enhanced the patient experience. This was collected on a simple colour-based three-point scale, given the low-literacy levels of our target population.

3. Quality of care to measure the effectiveness of curated training on nurses' performance and check the quality of medical services delivered.

D.3 Results and key learnings from the pilot

The first phase of the pilot ran in 3 clinics for 138 days, over 15 months, with 735 consultations and 832 registrations provided. Access was expanded to a population of 15,000 in this pilot itself. Overall, the pilot ran for about 2 years. In the pilot, the set-up cost for each clinic was about $4000, with an operating cost which ranged from $1000-$2000. The operating cost did not include the salaries of ANMs or the cost of medicines, which were paid for by existing government budgets. At scale, the set-up and the operating costs are likely to come down significantly.

Figure 13 gives a snapshot of the performance of the clinics on the key success metrics described above:

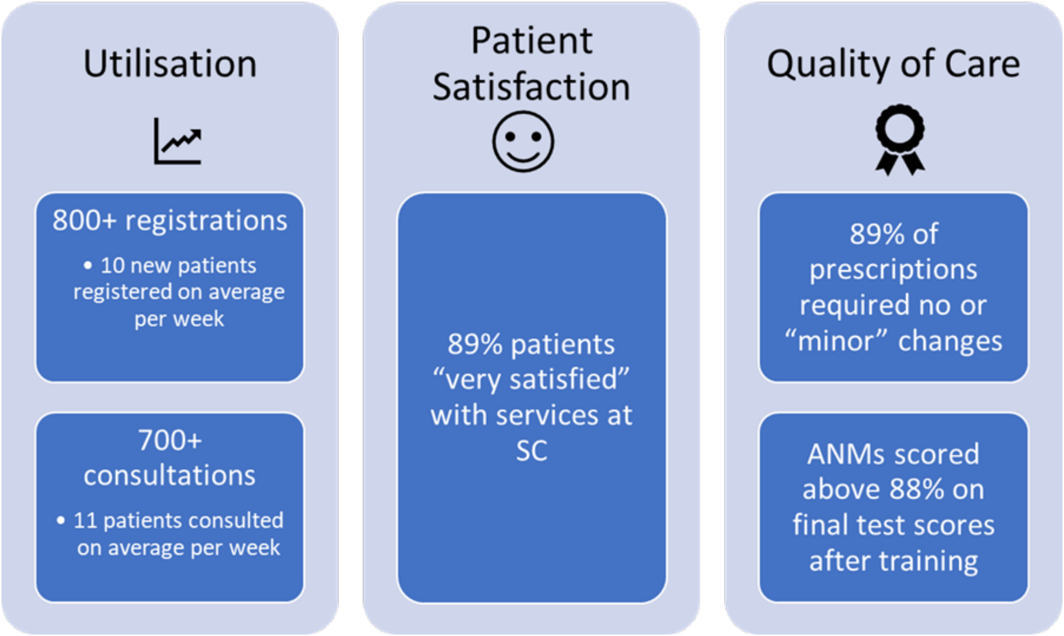

Figure 13: Overall Results from the Clinic Didi Model Pilot

In addition to the quantitative results, the pilot was able to generate multiple learnings for anyone who is trying out last-mile, nurse-led primary care clinics. All analysis has been done on entries in the electronic health records, which included all the required information. For example, when calculating the ages of patients registered, if no age or gender was recorded, the patient is not included in the analysis. The learnings from the pilot are discussed below.

D.3.1 ANMs can be trained to provide high-quality primary care in remote settings, and there is significant demand for such services

After a month of setting up, the three SHCs were seeing a steady footfall of 15-20 unique patient visits per week, with an average of 5 consultations every day. It was critical to know the population of registered patients as it helped in focusing the interventions on their needs. See, for example, the age distribution of the registered female and male patients across all the three SHCs in figures 14 and 15. Similarly, tracking the number of visits across weeks and days was helpful in understanding the best staffing and inventory needs on a regular basis (figure 16).

In the initial stages, there was a lot of enthusiasm in the local community regarding the new clinic in their village. The community previously had to travel 1-2 hours to get to the nearest government hospital for care. Post the initial excitement, the pilot saw footfalls of about 5 patients a day, with the total population covered rising from 5,000 to 20,000 from the beginning to the end of the pilot. Typically, the clinics were operational for four days a week, two hours per day. The footfall would be considerably higher if the clinics were operational for a standard of 6-8 hours a day (approximately 80-100 patients per day). However, this proved nearly impossible to implement within the government-run SHCs and the staff available there.

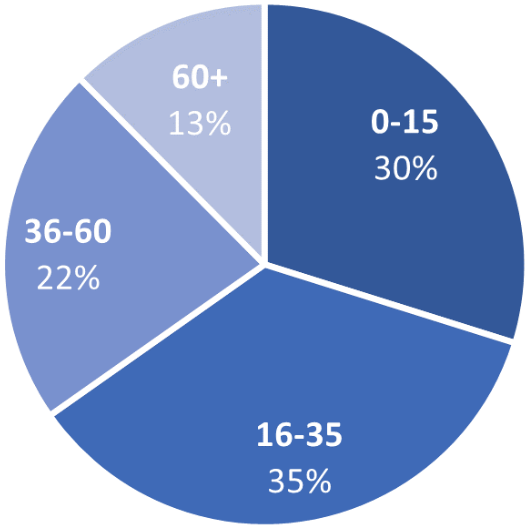

Figure 14: Age Distribution of Female Registered Patients (n=305)

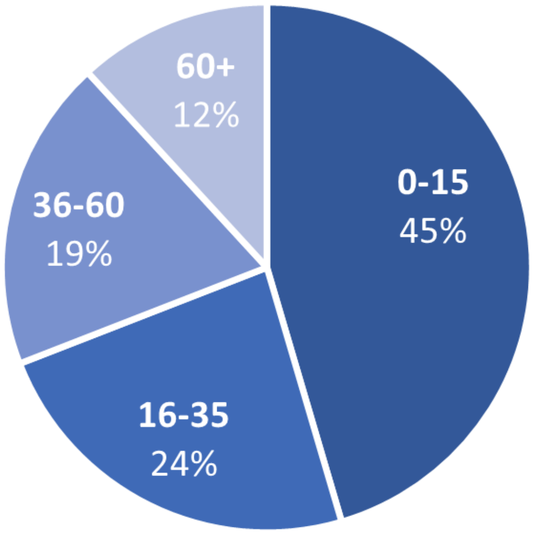

Figure 15: Age Distribution of Male Registered Patients (n=220)

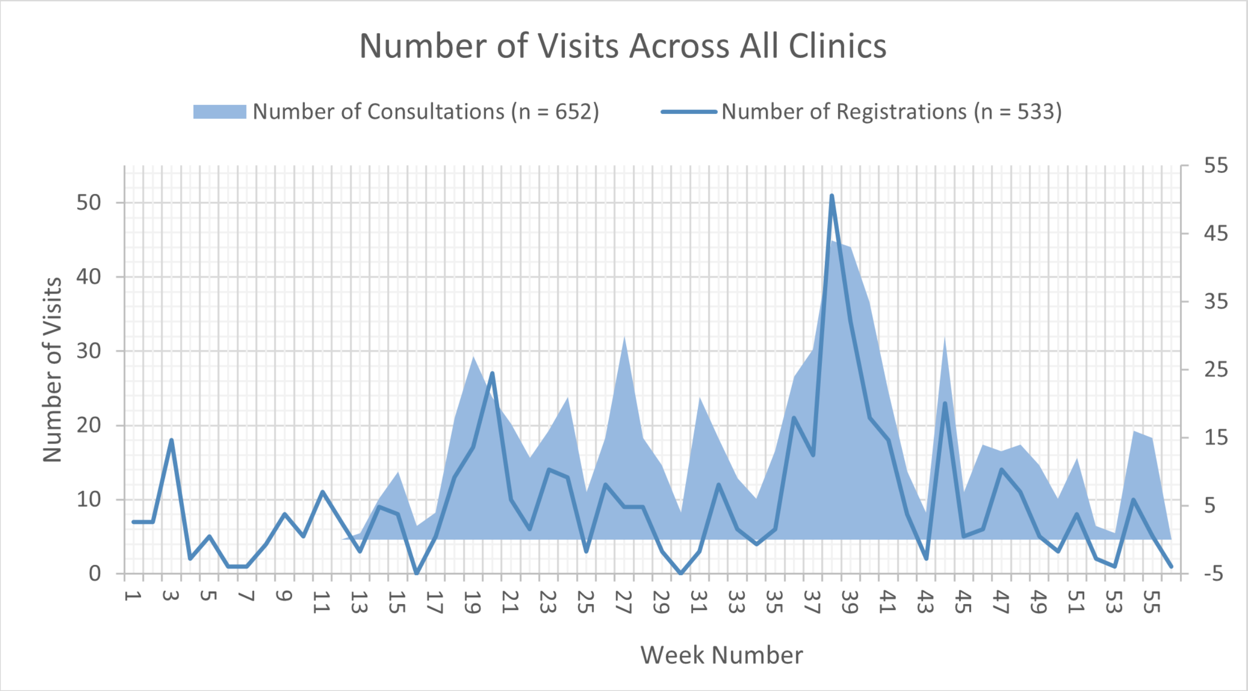

Figure 16: Number of consultations and registrations per week

By understanding why patients come in (figure 17), we could best equip the ANMs with training to look out for and deal with certain symptoms and ensure the clinic had the necessary drugs in stock. Furthermore, it painted a picture of why patients felt the need to come in. Since over 50% of the patients were coming, in all likelihood, for a seasonal cough, cold, or fever, we could ensure that the ANMs were always checking for this while also providing other preventative care.

In 2017, when the programme was implemented, there were very few targeted Electronic Health Record (EHR) platforms for ANM-led clinics. The programme created a simple EHR platform, completely customised for a rural ANM-led clinic with modules for patient and clinic management. The patient management module allowed the ANM to track patient details based on biometric identification (AADHAR-linked), run consultations, generate prescriptions, and make referrals, while the clinic management module allowed for inventory tracking (clinical supplies, drugs, etc.), clinic operations checklists, and attendance of staff. This application was developed on the Dimagi CommCare platform and rigorously user-tested with ANMs before clinic operations commenced.

As the EHR was implemented, about 43% of entries (121/278) included demographic information, which was used for a better understanding of the patient population. To address this gap, community health and registration camps were organised to allow the local population to screen for basic conditions and register themselves at the clinic. About 57% of all diagnoses were for women between the age of 16-60. This could be because they were more frequently at home in the village and had more time to spare for healthcare or because of certain challenges associated with men and health-seeking behaviours (men don't want to be seen as unwell because, as daily-wage earners, they need to be rehired every day).

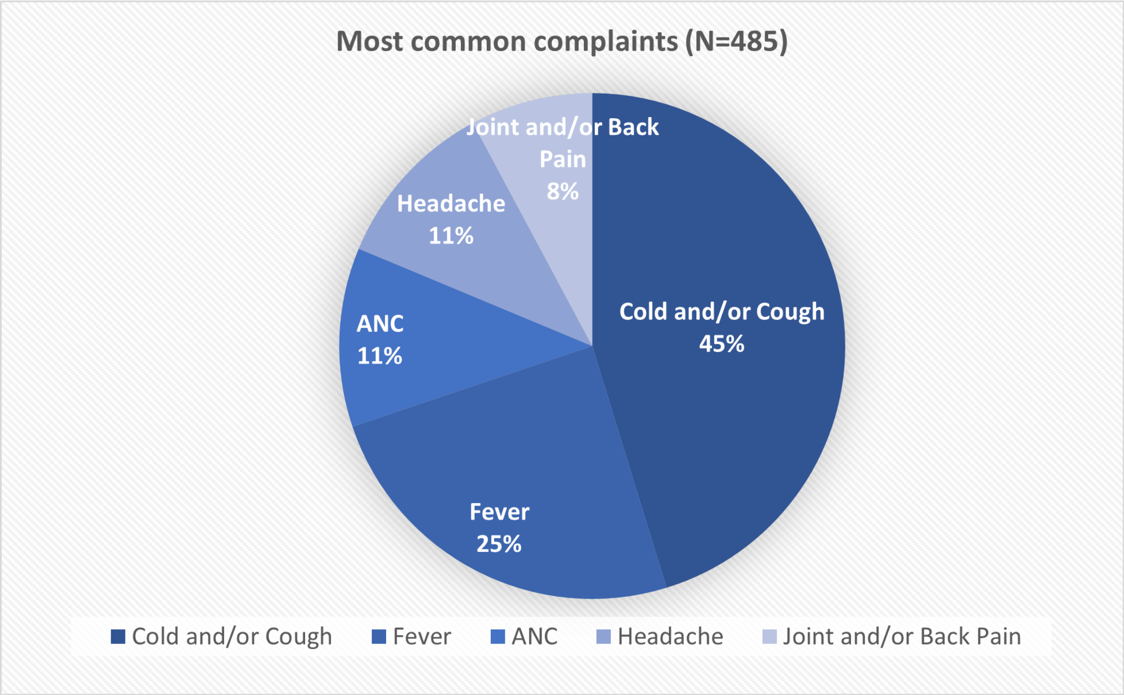

Figure 17: Most common overall complaints across all three clinics

	Time (mm:ss.mmm)	
	Registration	Consultation
Minimum	01:00.000	00:00.000
Maximum	11:00.000	48:00.000
Average	05:33.100	09:52.500
# of Patients (n)	297	735

Table 2: Wait times

D.3.2 Patients in rural areas, typically faced with challenges of long distances and wait times, valued timely care in a *one-stop-shop set up*

.

Over the one-year period of the pilot, about 89% of patients reported being "very satisfied" with the services at the clinic (figure 18). Patients mentioned the availability of diagnostic services, the availability of drugs, and in-depth consultation with the ANM as positives. The detailed health forms were particularly appealing to them as they associated these with the quality of caregiving. Taking time away from daily life in order to visit a clinic is difficult for all patients; therefore, all services being available under one roof and in a timely manner was very useful to them. On average, it took about five minutes for registration and ten minutes for a consultation (table 2). Because of the streamlined EHR system and technologically empowered ANMs, more patients could be seen with lower wait times. This was especially important since the outpatient clinic was only open for a limited amount of time every day.

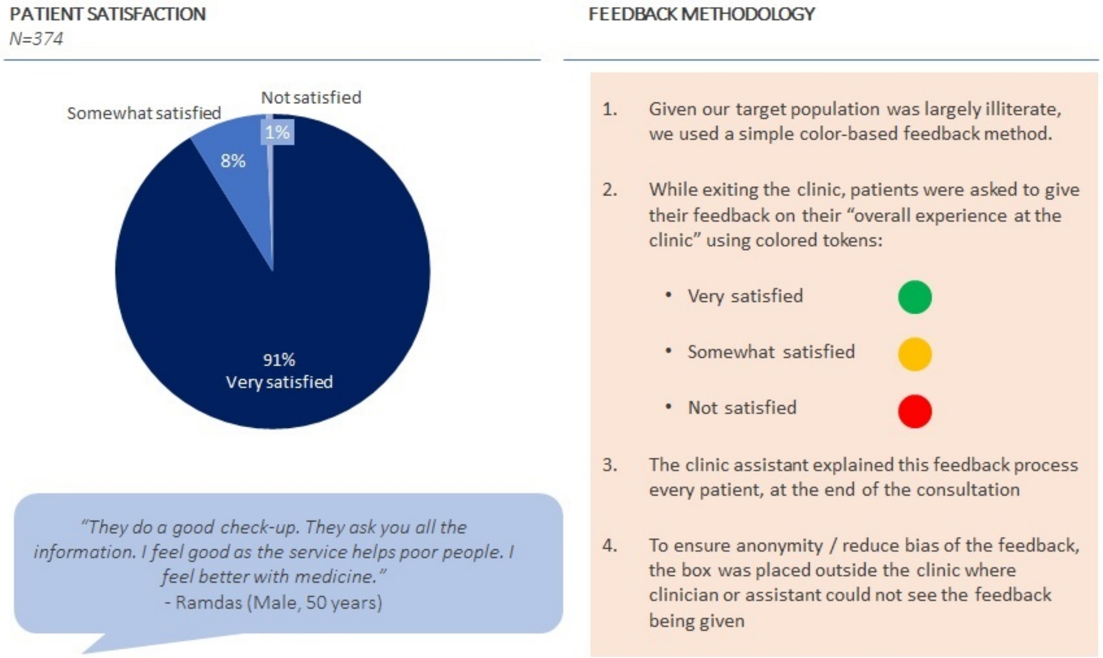

Figure 18: Patient satisfaction Indicators

Over a period, a number of challenges also arose, principally due to staff shortages across the entire block, resulting from ANMs being absent. This resulted in the clinic being non-operational for typically 4-5 days a month. During the pilot, it was also realised that there is a need for more regular patient engagement and counselling support to help combat common misconceptions, including the association of the use of intravenous drips or injections with receiving good medical treatment, a common practice amongst informal providers.

D.3.3 Digitization and training can dramatically help improve healthcare workers' skills and quality of care, but additional incentives and systemic changes are needed to improve attitudes and adherence

Quality of care was defined as the accuracy of ANMs' diagnoses and prescriptions, as well as their technical and clinical skills in dealing with primary care conditions. In the intervention period, there was a 40-60 per cent improvement in ANMs' clinical scores and technical skills (related to the use of EHR and POC devices). These were measured before, during, and after the course of the intervention.

In addition, 89% of the ANMs' prescriptions required minor or no changes, where minor changes were described as *No change to diagnosis, only 1-2 changes to medication or dosage* (figure 19). These were assessed via a prescription audit conducted by doctors both from government facilities, as well as from Clinic Didi's technical partner, SEWA Rural Hospital at Jhagadia in the Bharuch district of Gujarat. These were based on a random sample of her prescriptions generated over the first month of operations. In terms of clinical skills,

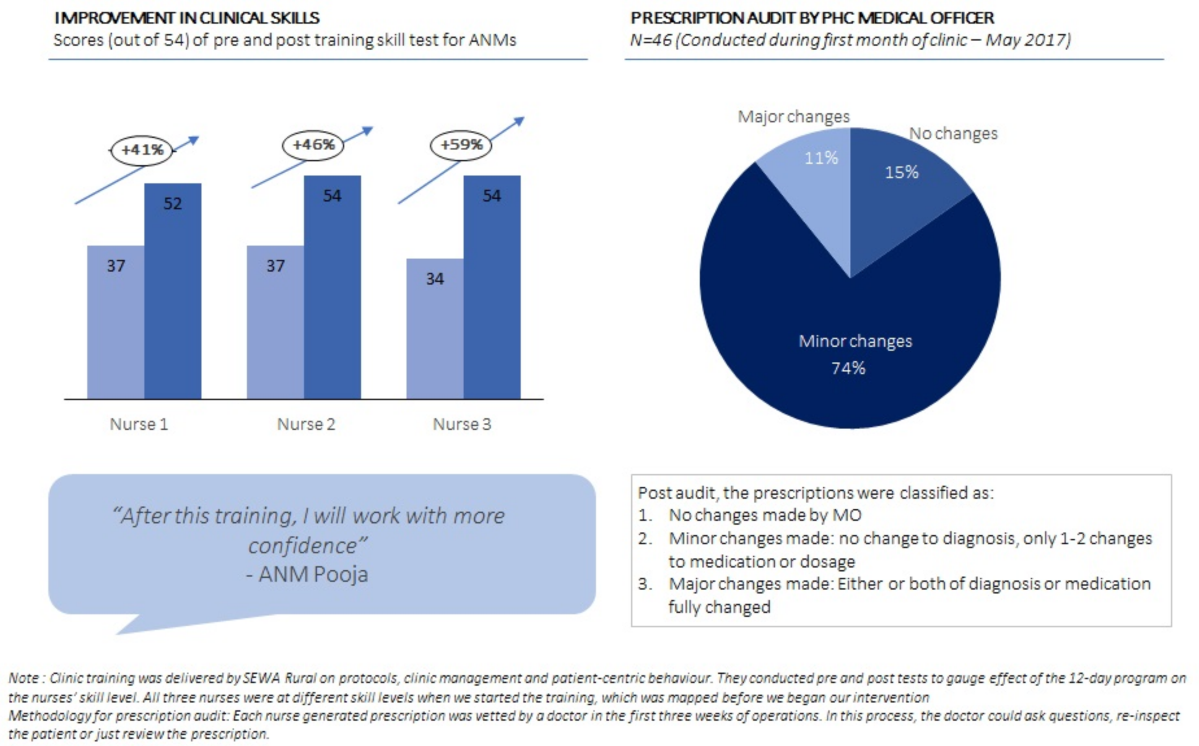

Figure 19: ANM Test Scores

before and after training examinations were conducted by SEWA Rural. The tests consisted of both practical and theory-based modules related to clinical protocols, medication and dosages and therapeutic treatment.

As can be seen from figure 19, over time, all scores have improved. However, despite these remarkable improvements in clinical and technical skills, nurses continue to be somewhat demotivated by the larger need for paper-based record keeping (which continues in the public sector across the country, despite the push for digitisation) for their other health program responsibilities, limited or no reward and recognition programs, and the perception of being overworked due to added responsibility of out-patient clinic, which in fact can be solved by better work-planning and scheduling. In addition to this, as mentioned above, because they are not locally recruited, most ANMs are posted away from their actual homes and families, which acts as a barrier to taking on more responsibilities.

References

1. Saprii L, Richards E, Kokho P, and Theobald S. Community health workers in rural India: analysing the opportunities and challenges Accredited Social Health Activists (ASHAs) face in realising their multiple roles. Human Resources for Health 2015;13.
2. Scott K, Javadi D, and Gergen J. India's Auxiliary Nurse-Midwife, Anganwadi Worker, Accredited Social Health Activist, Multipurpose Worker, and Lady Health Visitor Programs. 2018. URL: https://chwcentral.org/indias-auxiliary-nurse-midwife-anganwadi-worker-accredited-social-health-activist-multipurpose-worker-and-lady-health-visitor-programs/.
3. Werner D, Thuman C, and Maxwell J. Where There Is No Doctor (Hesperian Health Guides). Hesperian, 2022. URL: https://store.hesperian.org/prod/Where_There_Is_No_Doctor.html.
4. Lahariya C. Health & Wellness Centers to Strengthen Primary Health Care in India: Concept, Progress and Ways Forward. The Indian Journal of Pediatrics 2020;87:916–29.
5. IGNOU. Certificate in Community Health (BPCCHN/CCH). 2023. URL: http://www.ignou.ac.in/ignou/aboutignou/school/sohs/programmes/detail/674/2.
6. ACF. Ambuja Cement Foundation. 2020. URL: www.ambujacementfoundation.org.
7. Xu S and Hu D. Barefoot Doctors and the "Health Care Revolution" in Rural China: A Study Centered on Shandong Province. Endeavour 2017;41:136–45.
8. DGHS. Community Based Assessment Checklist (CBAC) Form for Early Detection of NCDs, Tuberculosis (TB) and Leprosy. 2016. URL: https://dghs.gov.in/WriteReadData/userfiles/file/Leprosy/LOCAL_REF_4964_1611895745717.pdf.
9. Joag K, Shields-Zeeman L, Kapadia-Kundu N, Kawade R, Balaji M, and Pathare S. Feasibility and acceptability of a novel community-based mental health intervention delivered by community volunteers in Maharashtra, India: the Atmiyata programme. BMC Psychiatry 2020;20:48.
10. MJS. Swachh Bharat Mission (Gramin). 2023. URL: https://swachhbharatmission.gov.in/sbmcms/index.htm.
11. SEC. Raksha Bandhan. 2017. URL: https://encyclopedia.pub/entry/29746.
12. IBRD. Leveraging Community Institutions to Support Agri-Business and Livelihoods in Jharkhand, India. Tech. rep. Washington, DC, USA: The World Bank, 2020:50. URL: http://documents.worldbank.org/curated/en/303211498576866109/India-Jharkhand-Opportunities-for-Harnessing-Rural-Growth-JOHAR-Project.
13. IBRD. Jharkhand's Pashu Sakhis: The Community Animal Healthcare Workers. 2022. URL: https://www.worldbank.org/en/news/feature/2022/12/13/jharkhand-s-pashu-sakhis-the-community-animal-healthcare-workers.
14. NHSRC. IPHS 2022 and its layouts. Tech. rep. New Delhi, India: National Health Systems Resource Centre, 2021. URL: https://nhsrcindia.org/IPHS2022.
